# The influence of embryo stage on obstetric complications and perinatal outcomes following programmed compared to natural frozen-thawed embryo transfer cycles: a systematic review and meta-analysis

**DOI:** 10.3389/fendo.2023.1186068

**Published:** 2023-08-16

**Authors:** Zhonghua Zhao, Yan Chen, Hongxia Deng, Lu Huang, Danhua Lu, Xiaoyang Shen, Liangzhi Xu

**Affiliations:** ^1^Department of Obstetrics and Gynecology, West China Second University Hospital, Sichuan University, Chengdu, China; ^2^Reproductive Endocrinology and Regulation Laboratory, West China Second University Hospital, Sichuan University, Chengdu, China; ^3^Key Laboratory of Birth Defects and Related Diseases of Women and Children, Ministry of Education, Sichuan University, Chengdu, China; ^4^The Joint Laboratory for Reproductive Medicine of Sichuan University, The Chinese University of Hong Kong, Chengdu, China

**Keywords:** frozen–thawed embryo transfer, programmed cycle, natural cycle, embryo, embryo at time of transfer

## Abstract

**Objective:**

To investigate the effect of embryo stage at the time of transfer on obstetric and perinatal outcomes in programmed frozen-thawed embryo transfer (FET) versus natural FET cycles.

**Design:**

Systematic review and meta-analysis.

**Setting:**

Not applicable.

**Patient(s):**

Women with programmed frozen-thawed embryo transfer (FET) and natural FET.

**Intervention(s):**

The PubMed, MEDLINE, and EMBASE databases and the Cochrane Central Register of Controlled Trials (CCRT) were searched from 1983 to October 2022. Twenty-three observational studies were included.

**Primary outcome measure:**

The primary outcomes were hypertensive disorders of pregnancy (HDPs), gestational hypertension and preeclampsia (PE). The secondary outcomes were gestational diabetes mellitus (GDM), placenta previa, postpartum haemorrhage (PPH), placental abruption, preterm premature rupture of membranes (PPROM), large for gestational age (LGA), small for gestational age (SGA), macrosomia, and preterm delivery (PTD).

**Result(s):**

The risk of HDP (14 studies, odds ratio (OR) 2.17; 95% confidence interval (CI) 1.95-2.41; P<0.00001; I^2^ = 43%), gestational hypertension (11 studies, OR 1.38; 95% CI 1.15-1.66; P=0.0006; I^2^ = 19%), PE (12 studies, OR 2.09; 95% CI 1.88-2.32; P<0.00001; I^2^ = 0%), GDM (20 studies, OR 1.09; 95% CI 1.02-1.17; P=0.02; I^2^ = 8%), LGA (18 studies, OR 1.11; 95% CI 1.07-1.15; P<0.00001; I^2^ = 46%), macrosomia (12 studies, OR 1.15; 95% CI 1.07-1.24; P=0.0002; I^2^ = 31%), PTD (22 studies, OR 1.21; 95% CI 1.15-1.27; P<0.00001; I^2^ = 49%), placenta previa (17 studies, OR 1.2; 95% CI 1.02-1.41; P=0.03; I^2^ = 11%), PPROM (9 studies, OR 1.19; 95% CI 1.02-1.39; P=0.02; I^2^ = 40%), and PPH (12 studies, OR 2.27; 95% CI 2.02-2.55; P <0.00001; I^2^ = 55%) were increased in programmed FET cycles versus natural FET cycles with overall embryo transfer. Blastocyst transfer had a higher risk of HDP (6 studies, OR 2.48; 95% CI 2.12-2.91; P<0.00001; I^2^ = 39%), gestational hypertension (5 studies, OR 1.87; 95% CI 1.27-2.75; P=0.002; I^2^ = 25%), PE (6 studies, OR 2.23; 95% CI 1.93-2.56; P<0.00001; I^2^ = 0%), GDM (10 studies, OR 1.13; 95% CI 1.04-1.23; P=0.005; I^2^ = 39%), LGA (6 studies, OR 1.14; 95% CI 1.07-1.21; P<0.0001; I^2^ = 9%), macrosomia (4 studies, OR 1.15; 95% CI 1.05-1.26; P<0.002; I^2^ = 68%), PTD (9 studies, OR 1.43; 95% CI 1.31-1.57; P<0.00001; I^2^ = 22%), PPH (6 studies, OR 1.92; 95% CI 1.46-2.51; P<0.00001; I^2^ = 55%), and PPROM (4 studies, OR 1.45; 95% CI 1.14-1.83; P=0.002; I^2^ = 46%) in programmed FET cycles than in natural FET cycles. Cleavage-stage embryo transfers revealed no difference in HDPs (1 study, OR 0.81; 95% CI 0.32-2.02; P=0.65; I^2^ not applicable), gestational hypertension (2 studies, OR 0.85; 95% CI 0.48-1.51; P=0.59; I^2^ = 0%), PE (1 study, OR 1.19; 95% CI 0.58-2.42; P=0.64; I^2^not applicable), GDM (3 study, OR 0.79; 95% CI 0.52-1.20; P=0.27; I^2^ = 21%), LGA (1 study, OR 1.15; 95% CI 0.62-2.11; P=0.66; I^2^not applicable), macrosomia (1 study, OR 1.22; 95% CI 0.54-2.77; P=0.64; I^2^ not applicable), PTD (2 studies, OR 1.05; 95% CI 0.74-1.49; P=0.79; I^2^ = 0%), PPH (1 study, OR 1.49; 95% CI 0.85-2.62; P=0.17; I^2^not applicable), or PPROM (2 studies, OR 0.74; 95% CI 0.46-1.21; P=0.23; I^2^ = 0%) between programmed FET cycles and natural FET cycles.

**Conclusion(s):**

The risks of HDPs, gestational hypertension, PE, GDM, LGA, macrosomia, SGA, PTD, placenta previa, PPROM, and PPH were increased in programmed FET cycles versus natural FET cycles with overall embryo transfer and blastocyst transfer, but the risks were not clear for cleavage-stage embryo transfer.

## Introduction

Frozen-thawed embryo transfer (FET) has increased dramatically since the first successful human pregnancy in 1983. This strategy enables the use of preimplantation genetic diagnosis/screening, facilitates fertility preservation, and reduces ovarian hyperstimulation syndrome in clinical practice (1). Compelling data have shown that FET results in a higher live birth rate than fresh embryo transfer. However, it was coupled with an increased risk for obstetric and perinatal complications (2). A meta-analysis performed by Roque et al. (2019) with 11 studies reported an increased risk of preeclampsia (PE) in pregnant women following FET compared with fresh ET (3). A pivotal multicentre RCT revealed a 3.13-fold increased risk of PE and a 1.6-fold increased risk of large for gestational age (LGA) in FET compared with fresh ET (4). To date, the reason FET leads to elevated obstetric and perinatal complications is unknown and possibly multifactorial. Recently, researchers proposed that the absence of the corpus luteum (CL) in the programmed endometrial preparation protocol, which is commonly used for FET, may be one potential contributor. Indeed, von Versen-Höynck et al. showed for the first time that the programmed FET cycle (0 CL) was associated with higher rates of preeclampsia and preeclampsia with severe features than the natural FET cycle (1 CL) (5). A meta-analysis including 9 studies reported a significant increase in hypertensive disorders of pregnancy (HDPs), PE, postpartum haemorrhage (PPH), placenta previa and preterm premature rupture of membranes (PPROM) following programmed FET cycles versus natural FET cycles (6). However, the abovementioned studies did not specify the embryo stage at the time of transfer, which may influence the pregnancy outcomes. Indeed, increased risks of preterm birth, LGA and perinatal mortality (7), as well as placenta-related diseases, including placenta previa, placental abruption and pregnancy-induced hypertension (8, 9), have been observed following blastocyst transfers versus cleavage-stage embryo transfers. However, another study reported that the embryo stage at transfer has a neutral effect on obstetric and perinatal outcomes (10). Therefore, we conducted a review and meta-analysis to compare the obstetric and perinatal outcomes of programmed FET cycles and natural FETs according to the type of embryo stage at the time of transfer (cleavage stage or blastocyst stage).

## Materials and methods

### Data sources and search strategy

An electronic search of literature was performed from 1983 to November 2022 in the database of PubMed, MEDLINE, EMBASE and Cochrane Central Register of Controlled Trials (CCRT), with the following search terms: (‘frozen embryo transfer’ OR ‘frozen-thawed embryo transfer’ OR ‘FET’ OR ‘vitrified-warmed embryo transfer’ OR ‘frozen blastocyst transfer’ OR ‘frozen cleavage-stage transfer’ OR ‘D5-6 frozen embryo transfer’ OR ‘D2-3 frozen embryo transfer’ OR ‘programmed frozen embryo transfer’ OR ‘natural frozen embryo transfer cycle’ OR ‘endometrial preparation protocols’ OR ‘hormone replacement therapy’ OR ‘artificial frozen-thawed embryo’ OR ‘corpus luteum’) AND (‘obstetric complication’ OR ‘pregnancy complication’ OR ‘perinatal complication’ OR ‘neonatal complication’ OR ‘preterm birth’ OR ‘gestational hypertension’ OR ‘preeclampsia’ OR ‘hypertensive disorders of pregnancy’ OR ‘Pregnancy induced hypertension’ OR ‘post-partum hemorrhage’ OR ‘placenta previa’ OR ‘placental abruption’ OR ‘post-term birth’ OR ‘gestational diabetes mellitus’ OR ‘premature rupture of membranes’ OR ‘macrosomia’ OR ‘large for gestational age’ OR ‘small for gestational age’). Articles in the reference lists that met the inclusion criteria were also searched manually.Twenty-three observational studies that comparing obstetric and/or perinatal outcomes between programmed FET cycles and natural FET cycles were included (Flow chart of studies).

### Criteria for inclusion and exclusion

Studies were included if they met the following criteria: (i) the study had at least two cohorts including programmed cycle FET versus natural FET cycle, and (ii) the study reported the obstetric and/or perinatal outcomes following programmed cycle FET versus natural FET cycle.

### Data extraction and quality assessment

Three authors independently screened each the title and abstract of each. Full-text articles were read if the study met the inclusion criteria. Then, they extracted data with a standard extraction form. Details for the data extraction are shown in **Table 1**. Three o other authors independently assessed the risk of bias of the included studies using the Newcastle-Ottawa Scale (NOS) in three domains: selection of study groups, comparability of groups and ascertainment of exposure. Detailed scores are shown in **Table 2**. A discussion was conducted with a third author if there was any disagreement.

**Table 1:** Description of included studies.

Study	Country	Design	Endometrial preparation	Embryo stage	FET protocol	Freezing technique	PGT	Age	Study quality
Asserhøj et al., 2021 (11)	Denmark	Register-based cohort study	NC-FET	Cleavage-stage Blastocyst	HCG trigger: no P4 support: no	Unclear	Exclude	34.5 (4.3)	7
			PC-FET		GnRH agonist use: +/ -Estradiol: Yes P4 support: yes			33.5 (4.4)	
Dallagiovanna et al., 2021 (12)	Italy	Retrospective cohort study	NC-FET	Blastocyst	HCG trigger: no P4 support: no	Unclear	A part of	36(33-38)	8
			PC-FET		GnRH agonist use: no Estradiol: Yes P4 support: yes			34 (31–37)	
Fu et al., 2022 (13)	China	Retrospective study	NC-FET	Blastocyst	HCG trigger: no P4 support: yes	Vitrification	All	31.83±4.28	8
			PC-FET		GnRH agonist use: no estradiol: Yes P4 support: yes			31.76±4.21	
Ginström Ernstad et al., 2019 (14)	Sweden	Retrospective cohort study	NC-FET	Cleavage-stage Blastocyst	HCG trigger: none P4 support: none	Vitrification and slow freezing	Unclear	34.9±4.1	7
			PC-FET		GnRH agonist use: +/- Route of estradiol: unclear P4 support: yes			34.3±4.3	
Guan et al., 2016 (15)	China	Retrospective cohort study	NC-FET	Cleavage-stage	HCG trigger: yes P4 support: none	Vitrification	Unclear	31.2 ± 5.0	6
			PC-FET		GnRH agonist use: no Route of estradiol: yes P4 support: yes			31.3 ± 5.2	
Gu et al., 2023 (16)	China	Multicenter retrospective cohort study	NC-FET	Blastocyst	HCG trigger: no P4 support: yes	Unclear	Exclude	32 (29, 35)	8
			PC-FET		GnRH agonist use: +/- Estradiol: yes P4 support: yes			32 (29,35)	
Hu et al., 2021 (17)	China	Retrospective cohort study	NC-FET	Blastocyst	HCG trigger: +/- P4 support: yes	Vitrification	Exclude	32.3 (4.1)	8
			PC-FET		GnRH agonist use: no Estradiol: yes P4 support: yes			31.5 (4.0)	
Jing et al., 2019 (18)	China	Retrospective cohort study	NC-FET	Cleavage-stage Blastocyst	HCG trigger: no P4 support: yes	Vitrification	Unclear	31 (28, 35)	8
			PC-FET		GnRH agonist use: no Estradiol: yes P4 support: yes			31 (28, 35)	
Li et al., 2021 (19)	China	Retrospective cohort study	NC-FET	Cleavage-stage Blastocyst	HCG trigger: no P4 support: no	Vitrification	Exclude	31.43 ± 3.74	6
			PC-FET		GnRH agonist use: no Estradiol: yes P4 support: yes			30.96 ± 3.70	
Li et al., 2022 (20)	China	Retrospective cohort study	NC-FET	Cleavage-stage Blastocyst	HCG trigger: yes P4 support: no	Vitrification	Exclude	31.0 (29.0–34.0)	7
			PC-FET		GnRH agonist use: no Estradiol: yes P4 support: ye			31.0 (29.0–34.0)	
Lin et al., 2020 (21)	China	Retrospective cohort study	NC-FET	Blastocyst	HCG trigger: +/- P4 support: yes	Vitrification	Unclear	28.74 ± 2.89	8
			PC-FET		GnRH agonist use: no Estradiol: yes P4 support: ye			29.08 ± 3.01	
Makhijani et al., 2020 (22)	USA	Retrospective cohort study	NC-FET	Blastocyst	HCG trigger: no P4 support: yes	Vitrification	Adjusted	35.0 ± 3.7	7
			PC-FET		GnRH agonist use: yes Estradiol: yes P4 support: ye			33.9 ± 3.9	
Man et al., 2021 (23)	China	Retrospective study	NC-FET	Blastocyst	HCG trigger: +/- P4 support: yes	Unclear	Unclear	29 (27-31)	8
			PC-FET		GnRH agonist use: no Estradiol: yes P4 support: ye			28 (26-31)	
Pan et al., 2020 (24)	China	Retrospective cohort study	NC-FET	Cleavage-stage	HCG trigger: no P4 support: yes	Vitrification	Unclear	28.49 ± 2.98	7
			PC-FET		GnRH agonist use: no Estradiol: yes P4 support: ye			28.18 ± 3.07	
Saito et al., 2017 (25)	Japan	Retrospective cohort study	NC-FET	Cleavage-stage Blastocyst	HCG trigger: unclear P4 support:unclear	Vitrified and slow-frozen embryo	Unclear	36.5 ± 3.7	6
			PC-FET		Unclear			35.3 ± 4.0	
Tatsumi et al., 2017 (26)	Japan	Retrospective cohort study	NC-FET	Cleavage-stage Blastocyst	Unclear	Not list	Unclear	37.9 (4.1)	8
			PC-FET		Unclear			36.6 (4.4)	
von Versen-Höynck et al., 2019 (5)	USA	Retrospective cohort study	NC-FET	Cleavage-stage Blastocyst	HCG trigger: yes P4 support: yes	Not list	Unclear	36.5±4.0	9
			PC-FET		GnRH agonist use: yes Estradiol: yes P4 support: yes			35.4±4.2	
Wang et al., 2020a (27)	China	Retrospective cohort study	NC-FET	Blastocyst	HCG trigger: yes P4 support: yes	Vitrification	Not clear	30.93 ± 4.16	8
			PC-FET		GnRH agonist use: no Estradiol: yes P4 support: yes			30.63 ± 4.14	
Wang et al., 2020b (28)	China	Retrospective cohort study	NC-FET	Cleavage-stage Blastocyst	HCG trigger: yes P4 support: yes	Vitrification	Exclude	32.51±4.26	8
			PC-FET		GnRH agonist use: no Estradiol: yes P4 support: yes			32.54±4.25	
Xu et al., 2022 (29)	China	Retrospective cohort study	NC-FET	Cleavage-stage Blastocyst	HCG trigger: no P4 support: yes	Vitrification	Exclude	32.68 ± 3.84	8
			PC-FET		GnRH agonist use: no Estradiol: yes P4 support: yes			31.66 ± 3.90	
Zaat et al., 2021 (30)	Netherlands	Retrospective analysis of a RCT	NC-FET	Cleavage-stage Blastocyst	HCG trigger: yes P4 support: yes	Not list	Not clear	33.3 ± 4.0	7
			PC-FET		GnRH agonist use: no Estradiol: yes P4 support: yes			33.8 ± 4.0	
Zhou et al., 2022 (31)	China	Retrospective cohort study	NC-FET	Cleavage-stage Blastocyst	HCG trigger: yes P4 support: yes	Vitrification	A part of	32.40±4.11	8
			PC-FET		GnRH agonist use: no Estradiol: yes P4 support: yes			32.43±4.49	
Zong et al., 2020 (32)	China	Retrospective cohort study	NC-FET	Blastocyst	HCG trigger: yes P4 support: yes	Vitrification	Exclude	30.5 ± 4.1	8
			PC-FET		GnRH agonist use: no Estradiol: yes P4 support: yes			30.8 ± 4.0	

Study quality was evaluated by the Newcastle-Ottawa Scale (NOS).

+/-, used in some patient but not all patients.

**Table 2:** Risk of bias and quality assessment.

	Selection				Comparability	Outcomes			
Study	Representativeness of the exposed cohort	Selection of the non-exposed cohort	Ascertainment of exposure	Outcome of interest was not present at the start of the study	Comparability of cohorts on the basis of the design or analysis	Assessment of outcome	Enough follow up	Adequacy of follow-up of cohorts	Total
Asserhøj et al., 2021 (11)	1	1	0	1	1	1	1	1	7
Dallagiovanna et al., 2021 (12)	1	1	1	1	1	1	1	1	8
Fu et al., 2022 (13)	1	1	1	1	1	1	1	1	8
Ginström Ernstad et al., 2019 (14)	1	1	0	1	1	1	1	1	7
Guan et al., 2016 (15)	1	1	1	1	0	0	1	1	6
Gu et al., 2023 (16)	1	1	1	1	1	1	1	1	8
Hu et al., 2021 (17)	1	1	1	1	1	1	1	1	8
Jing et al., 2019 (18)	1	1	1	1	1	1	1	1	8
Li et al., 2021 (19)	1	0	1	1	1	0	1	1	6
Li et al., 2022 (20)	1	1	1	1	1	1	1	1	7
Lin et al., 2020 (21)	1	1	1	1	1	1	1	1	8
Makhijani et al., 2020 (22)	1	1	0	1	1	1	1	1	7
Man et al., 2021 (23)	1	1	1	1	1	1	1	1	8
Pan et al., 2020 (24)	1	1	1	1	1	0	1	1	7
Saito et al., 2017 (25)	1	1	0	1	1	0	1	1	6
Tatsumi et al., 2017 (26)	1	1	1	1	1	1	1	1	8
von Versen-Höynck et al., 2019 (5)	1	1	1	1	2	1	1	1	9
Wang et al., 2020a (27)	1	1	1	1	1	1	1	1	8
Wang et al., 2020b (28)	1	1	1	1	1	1	1	1	8
Xu et al., 2022 (29)	1	1	1	1	1	1	1	1	8
Zaat et al., 2021 (30)	1	1	1	1	1	0	1	1	7
Zong et al., 2020 (32)	1	1	1	1	1	1	1	1	8

The quality of stdies was assessed using the Newcastle-Ottawa Scale scoring system.

### Outcome measures

The primary outcomes were hypertensive disorders of pregnancy (HDPs), gestational hypertension and preeclampsia. The secondary outcomes were as follows: gestational diabetes mellitus (GDM), placenta previa, postpartum haemorrhage (PPH), placental abruption, preterm premature rupture of membranes (PPROM), large for gestational age (LGA), small for gestational age (SGA), macrosomia, and preterm delivery.

### Statistical analyses

Statistical analyses were performed using Review Manager 5.3 (Nordic Cochrane Centre, Cochrane Collaboration). Dichotomous outcomes are presented as odds ratios (ORs) with 95% confidence intervals (CIs). We used the Mantel-Haenszel method and fixed-effects model to estimate the pooled effect of variables. Heterogeneity was assessed by the I-squared statistic (*I^2^*). When *I^2^* > 50%, sensitivity analysis was applied to identify the sources of heterogeneity by excluding studies one by one. Differences were considered significant at *P* < 0.05.

## Results

### Study characteristics

The initial literature search identified a total of 3788 potentially relevant publications. After the titles and abstracts were thoroughly screened by two investigators independently, the full-text articles of 126 potential studies were selected for further review. Finally, 23 retrospective studies that met the inclusion criteria were included in this meta-analysis. The flow diagram of the selection procedure is presented in the flow chart of studies. The study characteristics are detailed in **Table 1**. The overall outcomes in the current study are presented in **Table 3**.

**Table 3:** Results of the overall outcomes comparing natural and programmed cycle FET according to embryo stage at the time of transfer.

Outcome	Overall embryo transfer			Blastocyst transfer			Cleavage stage embryo transfers		
	No. of study	OR	GRADE	No. of study	OR	GRADE	No. of study	OR	GRADE
HDP	14	2.17 (1.95-2.41)	⊕⊕OO Low	6	2.48 (2.12-2.91)	⊕⊕OO Low	1	0.81 (0.32-2.02)	⊕OOO Very low
PE	11	2.15 (1.94, 2.39)	⊕⊕OO Low	6	2.23 (1.93-2.56)	⊕⊕OO Low	1	1.19 (0.58-2.42)	⊕OOO Very low
Gestational hypertension	9	1.40 (1.15- 1.70)	⊕⊕OO Low	5	1.87 (1.27-2.75)	⊕⊕OO Low	2	0.85 (0.48-1.51)	⊕OOO Very low
GDM	18	1.11 (1.03-1.19)	⊕⊕OO Low	10	1.13 (1.04-1.23)	⊕⊕OO Low	3	0.79 (0.52-1.20)	⊕OOO Very low
LGA	18	1.11 (1.07-1.15)	⊕OOO Very low	6	1.14 (1.07-1.21)	⊕⊕OO Low	1	1.15 (0.62-2.11)	⊕OOO Very low
Macrosomia	13	1.14 (1.06-1.23)	⊕⊕OO Low	4	1.15 (1.05-1.26)	⊕⊕OO Low	1	1.22 (0.54-2.77)	⊕OOO Very low
PTD	22	1.21 (1.15-1.27)	⊕⊕OO Low	9	1.43 (1.31-1.57)	⊕⊕OO Low	2	1.05 (0.74, 1.49)	⊕OOO Very low
SGA	18	1.05 (0.99-1.11)	⊕OOO Very low	6	0.96(0.85-1.07)	⊕⊕OO Low	1	0.85 (0.49-1.47)	⊕OOO Very low
Placenta previa	15	1.30 (1.10-1.55)	⊕⊕OO Low	9	1.23 (0.98-1.54)	⊕⊕OO Low	1	0.59 (0.31-1.14)	⊕OOO; Very low
Placental abruption	9	1.23 (0.81-1.89)	⊕⊕OO Low	5	1.28 (0.72-2.28)	⊕OOO Very low	1	0.86 (0.10-7.42)	⊕OOO Very low
PPROM	7	1.22 (1.02-1.46)	⊕⊕OO Low	4	1.45 (1.14-1.83)	⊕⊕OO Low	2	0.74 (0.46-1.21)	⊕OOO Very low
PPH	10	2.00 (1.66-2.42)	⊕⊕OO Low	6	1.92(1.46-2.51)	⊕⊕OO Low	1	1.49 (0.85-2.62)	⊕OOO Very low

### Hypertensive disorders of pregnancy

**Figure 1:**
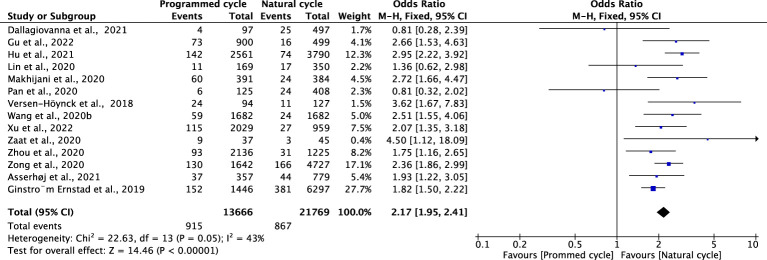
Hypertensive disorders of pregnancy.

Fourteen studies reported HDPs, including 21769 natural cycles and 13666 programmed cycles. The risk of HDP was significantly higher in programmed FET cycles (OR=2.17, 95% confidence interval (CI): 1.95-2.41, *P*<0.00001, *I^2^ =*43%). A subgroup analysis was performed to evaluate the effect of embryo stage at the time of transfer. With the use of blastocysts, there was an increased risk of HDP (OR 2.48, 95% CI 2.12-2.91, *P <*0.00001, *I^2^ =*39%) in programmed FET cycles compared with natural FET cycles. Only one study evaluated HDP for cleavage-stage embryo transfer and reported a non-significant increase in HDP in programmed FET cycles compared with natural FET cycles (OR=1.81, 95% CI: 0.32-2.02, *P* =0.65, *I^2^* not applicable) (**Figure 1**; **Supplementary Figure S1**).

### Gestational hypertension

**Figure 2:**
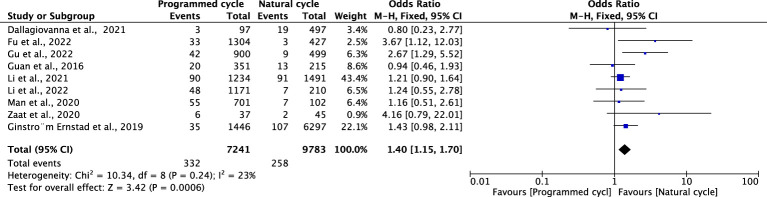
Gestational hypertention.

Nine studies including 9783 natural FET cycles and 7241 programmed FET cycles provided information on gestational hypertension. Compared with natural FET cycles, programmed FET cycles had a higher risk of gestational hypertension (OR 1.40, 95% CI 1.15-1.70, *P* =0.0006, *I^2^ =*23%). The subgroup analysis for blastocyst transfer revealed a higher risk of gestational hypertension in programmed cycles versus natural cycles (OR 1.87, 95% CI 1.27-2.75, *P* =0.002, *I^2^ =*25%). The subgroup analysis for cleavage-stage embryo transfer revealed no difference in gestational hypertension between programmed cycles and natural cycles (OR 0.85, 95% CI 0.48-1.51, *P* =0.59, *I^2^ =*0%) (**Figure 2**; **Supplementary Figure S2**).

### Preeclampsia

**Figure 3:**
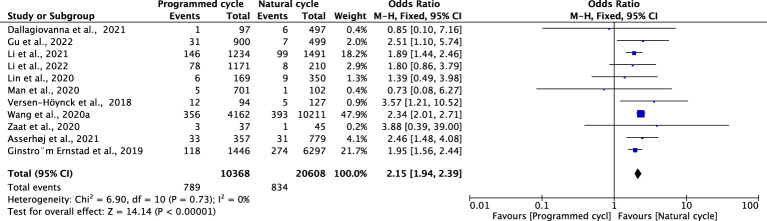
Preeclampsia.

Eleven studies with a total of 20608 natural FET cycles and 10368 programmed FET cycles were pooled. We observed a higher risk of preeclampsia in programmed cycles than in natural cycles (OR 2.15, 95% CI 1.94-2.39, *P <*0.00001, *I^2^ =*0%). The subgroup analysis revealed a higher risk of preeclampsia in programmed cycles than in natural cycles after blastocyst transfer (OR 2.23, 95% CI 1.93-2.56, *P <*0.00001, *I^2^ =*0%). Only one study evaluated preeclampsia for cleavage-stage embryo transfer, showing no difference between programmed cycles and natural cycles (OR=1.19, 95% CI: 0.58-2.42, *P* =0.64, *I^2^* not applicable) (**Figure 3**; **Supplementary Figure S3**).

### Gestational diabetes mellitus

**Figure 4:**
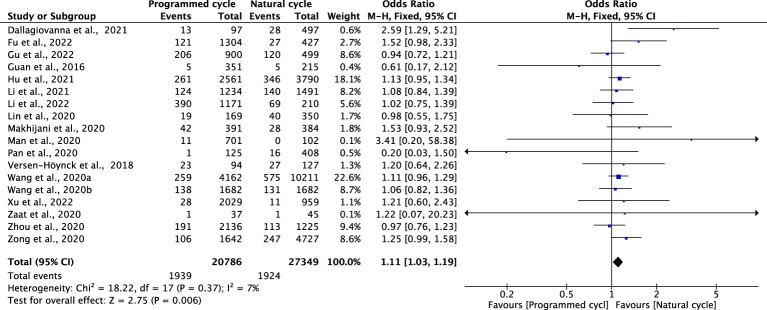
Gestational diabetes mellitus.

Eighteen studies reported GDM with 27349 natural FET cycles and 20768 programmed FET cycles. The incidence of GDM in programmed cycles was higher than that in natural cycles (OR 1.11, 95% CI 1.03-1.19, *P* =0.0006, *I^2^ =*7%). The subgroup analysis in patients with blastocyst transfer also revealed a higher risk of GDM in programmed cycles than natural cycles (OR 1.13, 95% CI 1.04-1.23, *P* =0.005, *I^2^ =*39%). However, the subgroup analysis for cleavage-stage embryo transfer revealed no difference in GDM between programmed cycles and natural cycles (OR 0.79, 95% CI 0.52-1.02, *P* =0.27, *I^2^ =*21%) (**Figure 4**; **Supplementary Figure S4**).

### Large for gestational age

**Figure 5:**
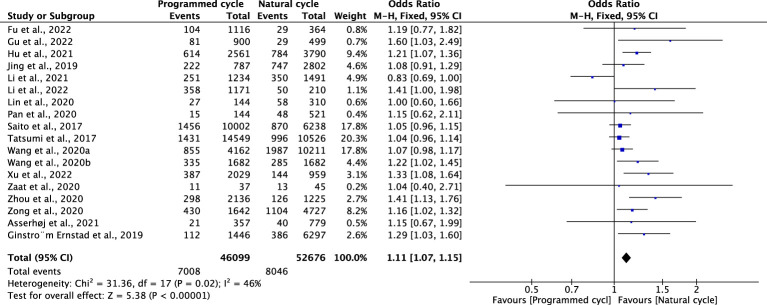
Large for gestational age.

Eighteen studies including 52676 natural cycles and 46099 programmed cycles provided information on LGA. A higher risk of LGA was observed in programmed cycles when compared with natural cycles (OR 1.11, 95% CI 1.07-1.15, *P <*0.00001, *I^2^ =*46%). In the subgroup analysis for blastocyst transfer, there was an increased risk of LGA in programmed cycles versus natural cycles (OR 1.14, 95% CI 1.07-1.21, *P <*0.0001, *I^2^ =*9%). 1.19). However, for cleavage-stage embryo transfer, no difference in LGA was found between programmed cycles and natural cycles in one study (OR 1.15, 95% CI 0.62-2.11, *P* =0.66, *I^2^* not applicable) (**Figure 5**; **Supplementary Figure S5**).

### Macrosomia

**Figure 6:**
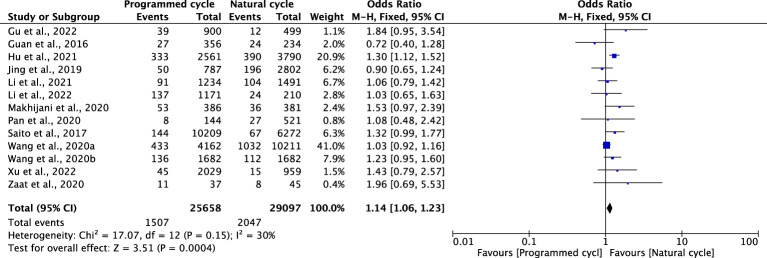
Macrosomia.

Thirteen studies that included 29097 natural FET cycles and 25658 programmed FET cycles evaluated macrosomia. The risk of macrosomia was significantly higher in programmed cycles than in natural cycles (OR 1.14, 95% CI 1.06-1.23, *P* =0.0004, *I^2^ =*30%). For blastocyst transfer, there was an increased risk of macrosomia in programmed cycles versus natural cycles (OR 1.15, 95% CI 1.05-1.26, *P* =0.002 *I^2^ =*68%). Only one study evaluated macrosomia following cleavage-stage embryo transfer and reported no difference in macrosomia between programmed cycles and natural cycles (OR=1.22, 95% CI: 0.54-2.77, *P* =0.64, *I^2^* not applicable) (**Figure 6**; **Supplementary Figure S6**).

### Secondary outcomes

#### Preterm delivery

**Figure 7:**
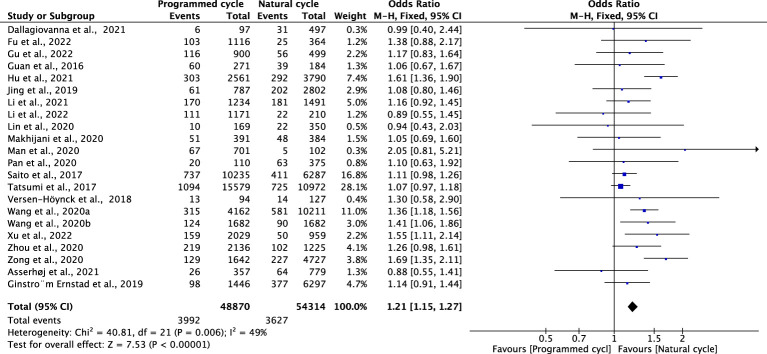
Preterm delivery.

Two studies, including 54314 natural FET cycles and 48870 programmed FET cycles, reported preterm delivery. The pooled result showed that the overall risk of PTD was significantly higher in programmed cycles than in natural cycles (OR 1.21, 95% CI 1.15-1.27, *P <*0.00001, *I^2^ =*49%). The subgroup analysis for blastocyst transfer revealed an elevated risk of GDM in programmed cycles versus natural cycles (OR 1.43, 95% CI 1.31-1.57, *P <*0.00001, *I^2^ =*22%). However, the subgroup analysis for cleavage-stage embryo transfer revealed no difference in GDM between programmed cycles and natural cycles (OR 1.05, 95% CI 0.74-1.49, *P* =0.96, *I^2^ =*0%) (**Figure 7**; **Supplementary Figure S7**).

### Small for gestational age

**Figure 8:**
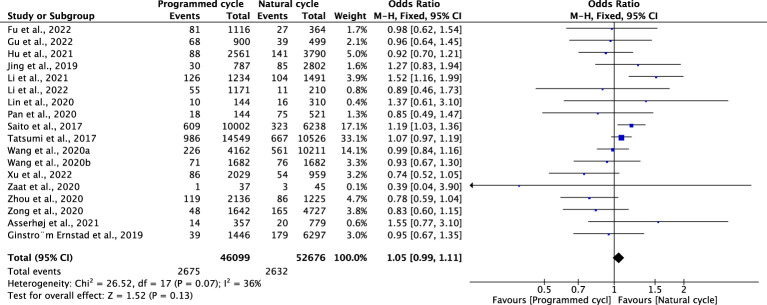
Small for gestational age.

Eighteen studies, which included 52676 natural FET cycles and 46099 programmed FET cycles, were meta-analysed. There was no difference in the incidence of SGA between natural cycles and programmed cycles (OR 1.05, 95% CI 0.99-1.11, *P* =0.13, *I^2^ =*36%). The results of the subgroup analyses revealed that the incidence of SGA in blastocyst transfer (OR 0.96, 95% CI 0.85-1.07, *P* =0.07, *I^2^ =*36%) and cleavage-stage embryo transfer (OR 0.85, 95% CI 0.49-1.47, *P* =0.56, *I^2^* not applicable) were consistent with the overall results (**Figure 8**; **Supplementary Figure S8**)

### Placenta previa

**Figure 9:**
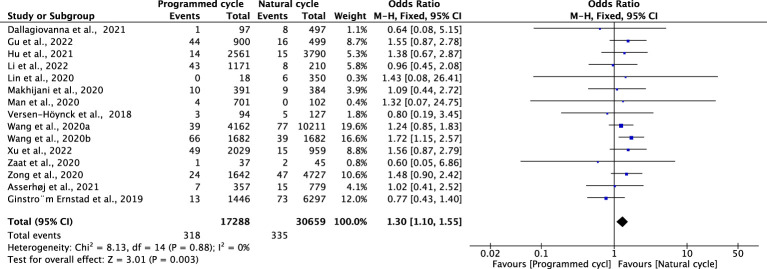
Placenta previa.

Fifteen studies were included in the analysis (programmed cycles= 17288; natural cycles =30659). The overall risk of placenta previa was significantly higher in pregnancies resulting from programmed cycles (OR 1.30, 95% CI 1.10-1.55, *P* =0.003, *I^2^ =*0%). The subgroup analyses revealed no difference in placenta previa between programmed cycles and natural cycles in either blastocyst transfer (OR 1.23, 95% CI 0.98-1.54, *P* =0.60, *I^2^ =*0%) or cleavage-stage embryo transfer (OR 0.59, 95% CI 0.31-1.14, *P* =0.12, *I^2^* not applicable) (**Figure 9**; **Supplementary Figure S9**).

### Placental abruption

**Figure 10:**
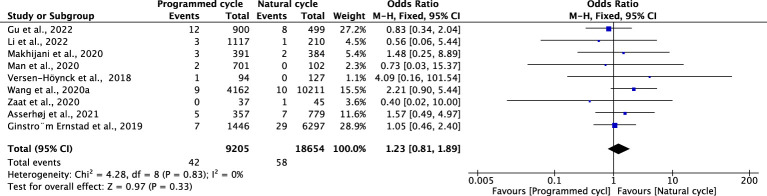
Placental abruption.

Fifteen studies were included in the analysis (programmed cycles= 9205; natural cycles =18654). The overall risk of placental abruption was similar between programmed FET cycles and natural FET cycles (OR 1.23, 95% CI 0.81-1.89, *P* =0.33, *I^2^ =*0%). The results of the subgroup analyses for blastocyst transfer (OR 1.28, 95% CI 0.72-2.28, P=0.4, *I^2^ =*0%) and cleavage-stage embryo transfer (OR 0.86, 95% CI 0.10-7.42, *P* =0.89, *I^2^* not applicable) were consistent with the overall results (**Figure 10**; **Supplementary Figure S10**).

### Preterm premature rupture of membranes

**Figure 11:**
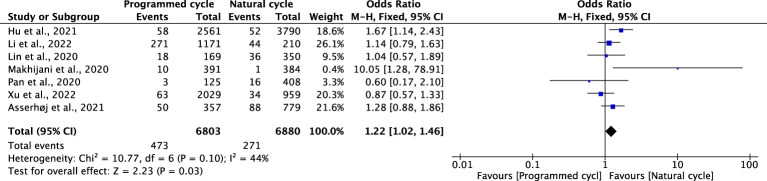
Preterm premature rupture of membranes.

Seven studies reported PPROM, including 6880 natural cycles and 6803 programmed cycles. The overall risk of PPROM was higher among the pregnancies resulting from the programmed FET cycles (OR 1.22, 95% CI 1.02-1.46, *P* =0.03, *I^2^ =*44%). The subgroup analysis for blastocyst transfer was consistent with the overall results (OR 1.45, 95% CI 1.14-1.83, *P* =0.002, *I^2^ =*46%). However, cleavage-stage embryo transfer showed no difference in PPROM between programmed cycles and natural cycles (OR=0.74, 95% CI: 0.46-1.21, *P* =0.23, *I^2^ =*0%) (**Figure 11**; **Supplementary Figure S11**).

### Postpartum haemorrhage

**Figure 12:**
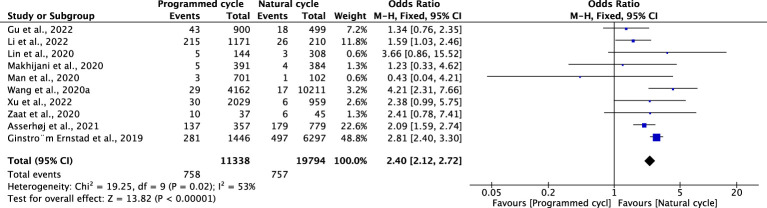
Post-partum hemorrhage.

Ten studies reported PPH, including 11338 natural cycles and 19794 programmed cycles. The overall risk of PPH was higher in the pregnancies resulting from the programmed FET cycles than in those resulting from natural FET cycles (OR 2.40, 95% CI 2.12-2.72, *P <*0.00001, *I^2^ =*53%). After a study suspected of being the source of heterogeneity was excluded (14), sensitivity analysis revealed that programmed cycles still had a significantly higher risk of PPH (OR 2.00, 95% CI 1.66-2.42, *P <*0.00001, *I^2^ =*34%). The subgroup analysis for blastocyst transfer was consistent with the overall results (OR 1.92, 95% CI 1.46-2.51, *P <*0.00001, *I^2^ =*55%). However, the subgroup analysis for cleavage-stage embryo transfer showed no difference in PPH between programmed cycles and natural cycles (OR 1.49, 95% CI: 0.85-2.62, *P* =0.17, *I^2^* not applicable) (**Figure 12**; **Supplementary Figure S12**).

## Discussion

In our analysis of obstetric and perinatal outcomes between natural FET cycles and programmed FET cycles, we showed that programmed cycles had a higher risk of HDPs, GH, PE, GDM, LGA, macrosomia, PD, PP, PPROM and PPH than natural cycles, which is consistent with the meta-analysis (6). Similar results were also achieved in another meta-analysis (33). Via subgroup analysis, our present meta-analysis further evaluated the effect of embryo stage at the time of transfer on perinatal outcomes between programmed FET cycles and natural FET cycles and showed that the perinatal outcomes of blastocyst transfer were consistent with the overall results in programmed FET cycles; that is, the incidences of HDP, GH, PE, PPROM and PPH were elevated in programmed FET cycles compared with natural FET cycles following blastocyst transfer. However, the perinatal outcomes of cleavage-stage embryo transfers in programmed FET cycles are similar to those in natural FET cycles.

To date, studies evaluating the effect of cleavage-stage embryo and blastocyst transfers on pregnancy outcomes have shown conflicting results. Ginström Ernstad et al. reported that women with blastocyst transfer had a 2.08-fold increased risk of placenta previa and a 1.62-fold increased risk of placental abruption versus cleavage-stage embryo transferss (34). In contrast, a meta-analysis performed by Rosalik et al. including 15 studies reported that both blastocyst transfers and cleavage-stage transfers could lead to a higher risk of LGA in programmed FET cycles versus natural FET cycles (35). Our present study finds a neutral effect for cleavage-stage transfers on obstetric and perinatal outcomes in programmed FET cycles versus natural FET cycles. Of note, the number of studies included here for cleavage-stage embryo transfer were too small to obtain reliable results, so these findings should be considered with great caution.

The reason for the higher risk of HDPs in the programmed FET cycles is unknown. The role of the corpus luteum has recently been a focus of attention for investigators. Indeed, a prospective cohort study of singleton pregnancy reported that women with 0 CL had elevated rates of PE (12.8% versus 3.9%; P=0.02) and PE with severe features (9.6% versus 0.8%; P=0.002) compared to those who conceived with 1 CL. After adjusting for confounders, 0 CL was shown to be a positive predictor of PE and sPE (5). Similar results were also achieved in another prospective study (35). Recently, a hypothesis was proposed that the absence of the CL in programmed cycles may lead to impaired maternal haemodynamic and cardiovascular adaptation to pregnancy in the first trimester, which is associated with adverse pregnancy outcomes such as preeclampsia (36). Compelling findings support the hypothesis that the expected decline in carotid-femoral pulse wave velocity (cfPWV) and the expected rise in transit time (cfPWTT) in the first trimester are attenuated in women with 0 CL (programmed FET cycle) relative to normal women, which suggests that arterial compliance is impaired in pregnant women with no CL (37). Moreover, pregnant women with 0 CL also have a blunted reactive hyperaemia index, an index that reflects endothelial function in early pregnancy, when compared to women with 1 CL (37). All these observations have prompted investigators to explore which factors are secreted into the circulation of women by the CL that may be important for maternal cardiovascular adaptation to pregnancy. Relaxin, a 6-kDa peptide, is probably a potential mediator (38). It is predominantly secreted by CL granulosa lutein cells in the late luteal phase (50-100 pg/ml) and reaches a peak concentration of 1000-2500 pg/mL in the first trimester when pregnancy occurs (39). Circulating relaxin was undetectable in women lacking CL (40, 41). It binds to membrane-associated relaxin family peptide receptor 1 (RXFP1), which is a G protein-coupled receptor that is widely distributed in the uterus (myometrium and epithelial layer), ovary and placenta (42) and vascular smooth muscle (unpublished data). Relaxin is a potent vasodilator that mediates vasodilation through the activation of Gi/PI3K-induced cAMP and nitric oxide synthase (nNOS)-driven NO release (43). An experimental study showed that systemic and renal vasodilation and global arterial compliance in early pregnancy were decreased, which contributed to lower cardiac output and a lower glomerular filtration rate when a relaxin-neutralizing antibody was administered to gravid rats (44–46). In humans, the expected rise in 24-hour creatinine clearance was blunted in pregnant women with ovum donation (no circulating relaxin) compared with normal pregnant women, which suggests that the renal response is also impaired in human pregnancy (8, 41). Therefore, relaxin could play a similar role in the maternal circulatory changes that occur during the first trimester. Relaxin deficiency (0 CL) is a potential compromise of maternal cardiovascular adaptation to pregnancy, which is the basic circulatory pattern that occurs in preeclampsia. Indeed, Post Uiterweer et al. found that women with low relaxin concentrations (lowest centile: < p10) during the first trimester are at increased risk of developing late-onset preeclampsia (36). In addition, women with donor-fresh and donor-thawed treatment have significantly higher odds of HDPs relative to women undergoing autologous-fresh treatment. A common feature among donor oocyte cycles is the usage of programmed endometrial preparation, which has no functioning CL (47). Most strikingly, relaxin has been recommended as a potential therapeutic candidate for preeclampsia (38). On the other hand, relaxin is a potent stimulus of endometrial maturation (decidualization), which governs trophoblast invasion during pregnancy (48). Relaxin can enhance the effect of progestin on the induction of prolactin and insulin growth factor binding protein-1 (IGFBP-1) secretion as well as glycodelin expression from human endometrial stromal cells (hESCs), which are biomarkers of decidualization (42, 49). In an exogenous oestradiol- and progesterone-treated ovariectomized rhesus monkey model, giving relaxin can increase its resident endometrial lymphocyte number and promote endometrial angiogenesis compared to controls (50, 51). Therefore, deficient CL-derived relaxin in early pregnancy probably contributes to aberrant decidualization, which is an important contributor to downregulated cytotrophoblast invasion, impaired placentation and consequently the genesis of placenta-related diseases, such as HDPs, placenta previa, PPROM and PPH (33, 52). The conventional theory is that preeclampsia causes impaired trophoblast invasion and uterine spiral artery remodelling, which can lead to impaired placentation, including reduced placental perfusion and placental ischaemia and reperfusion injury. In addition to CL, suboptimal steroid hormone administration in the programmed FET cycle may also affect trophoblast invasion through immunomodulation in the first trimester (48) and, as a result, influence placenta formation and function. For example, oestrogen has different effects on immune modulation depending on its concentration. Natural killer cells, which have been considered prime regulators of trophoblast invasion, are inhibited by pregnancy levels of oestradiol (E2) but are stimulated at dioestrus to proestrus levels of E2. IL-1 is stimulated by E2 at low concentrations but is inhibited by E2 at pregnancy levels. In addition, IL-6 is inhibited by E2 at periovulatory to pregnancy levels with no effects at early follicular or postmenopausal levels (53). Interestingly, these interleukins have been reported to play a role in trophoblast invasion during early pregnancy, which is involved in the genesis of HDPs. Indeed, Albrecht et al. found that elevated oestrogen levels in baboons lead to markedly decreased invasion of uterine spiral arteries by placental extravillous cytotrophoblasts, an important cell type involved in maintaining placental function (54). To date, we know little about the specific mechanism underlying the association between endometrial preparation for ET and adverse obstetric and neonatal complications.

## Limitations

Our study has several limitations. The first is that the included studies are retrospective in design, and there are inherent biases across them that we cannot address. Another limitation is that we included preimplantation genetic testing cycles. In addition, we did not specify the true natural FET and modified natural cycles.

## Conclusion

Our research showed that programmed FET cycles resulted in adverse obstetric and perinatal outcomes relative to natural FET cycles following mixed frozen embryo transfer (combined cleavage stage and blastocyst stage) or frozen blastocyst transfer, such as HDPs, gestational hypertension, PE, GDM,LGA, macrosomia, SGA, PTD, placenta previa, PPROM, and PPH. However, the obstetric and perinatal outcomes were similar after frozen cleavage-stage embryo transfers. Further investigations, including RCTs, should be conducted to elucidate the reason for obstetric and perinatal outcomes in programmed FET cycles.

## Data availability statement

The original contributions presented in the study are included in the article/**Supplementary Material**. Further inquiries can be directed to the corresponding author.

## Author contributions

Designer of the study: ZZ. Data acquisition and analysis: ZZ, YC, HD, XS, DL, LH. Draft of the manuscript and interpretation: ZZ. Revision of the manuscript: LX. All authors contributed to the article and approved the submitted version.
