# Supplementary material for: The influence of embryo stage on obstetric complications and perinatal outcomes following programmed compared to natural frozen-thawed embryo transfer cycles: a systematic review and meta-analysis
Source: Front Endocrinol (Lausanne). 2023 Aug 16;14:1186068. doi: 10.3389/fendo.2023.1186068 (PMC10468995; doi:10.3389/fendo.2023.1186068)

Obstetric complications and perinatal outcomes in programmed frozen embryo transfer (FET)cycles versus natural FET cycles:effects of embryo stage at time of transfer.

	Programmed cycle		Natural cycle			Odds Ratio
**Study or Subgroup**	**Events**	**Total**	**Events**	**Total**	**Weight**	**M-H, Fixed, 95% CI**
**1.1.1 Blastocyst Transfer**						
Dallagiovanna et al., 2021	4	97	25	497	1.7%	0.81 [0.28, 2.39]
Gu et al., 2022	73	900	16	499	4.1%	2.66 [1.53, 4.63]
Hu et al., 2021	142	2561	74	3790	12.3%	2.95 [2.22, 3.92]
Lin et al., 2020	11	169	17	350	2.2%	1.36 [0.62, 2.98]
Makhijani et al., 2020	60	391	24	384	4.5%	2.72 [1.66, 4.47]
Zong et al., 2020	130	1642	166	4727	17.1%	2.36 [1.86, 2.99]
**Subtotal (95% CI)**		**5760**		**10247**	**41.9%**	**2.48 [2.12, 2.91]**
Total events	420		322			
Heterogeneity: $Chi^2 = 8.13, df = 5 (P = 0.15); I^2 = 39\%$						
Test for overall effect: Z = 11.36 (P < 0.00001)						
**1.1.2 Cleavage-stage Embryo Transfer**						
Pan et al., 2020	6	125	24	408	2.3%	0.81 [0.32, 2.02]
**Subtotal (95% CI)**		**125**		**408**	**2.3%**	**0.81 [0.32, 2.02]**
Total events	6		24			
Heterogeneity: Not applicable						
Test for overall effect: Z = 0.46 (P = 0.65)						
**1.1.3 Not separated**						
Versen-Höynck et al., 2018	24	94	11	127	1.5%	3.62 [1.67, 7.83]
Wang et al., 2020b	59	1682	24	1682	5.0%	2.51 [1.55, 4.06]
Xu et al., 2022	115	2029	27	959	7.5%	2.07 [1.35, 3.18]
Zaat et al., 2020	9	37	3	45	0.4%	4.50 [1.12, 18.09]
Zhou et al., 2020	93	2136	31	1225	8.2%	1.75 [1.16, 2.65]
Asserhøj et al., 2021	37	357	44	779	5.4%	1.93 [1.22, 3.05]
Ginstro¨m Ernstad et al., 2019	152	1446	381	6297	27.7%	1.82 [1.50, 2.22]
**Subtotal (95% CI)**		**7781**		**11114**	**55.8%**	**1.99 [1.72, 2.30]**
Total events	489		521			
Heterogeneity: $Chi^2 = 5.68, df = 6 (P = 0.46); I^2 = 0\%$						
Test for overall effect: Z = 9.38 (P < 0.00001)						
**Total (95% CI)**		**13666**		**21769**	**100.0%**	**2.17 [1.95, 2.41]**
Total events	915		867			
Heterogeneity: $Chi^2 = 22.63, df = 13 (P = 0.05); I^2 = 43\%$						
Test for overall effect: Z = 14.46 (P < 0.00001)						
Test for subgroup differences: $Chi^2 = 8.72, df = 2 (P = 0.01), I^2 = 77.1\%$						

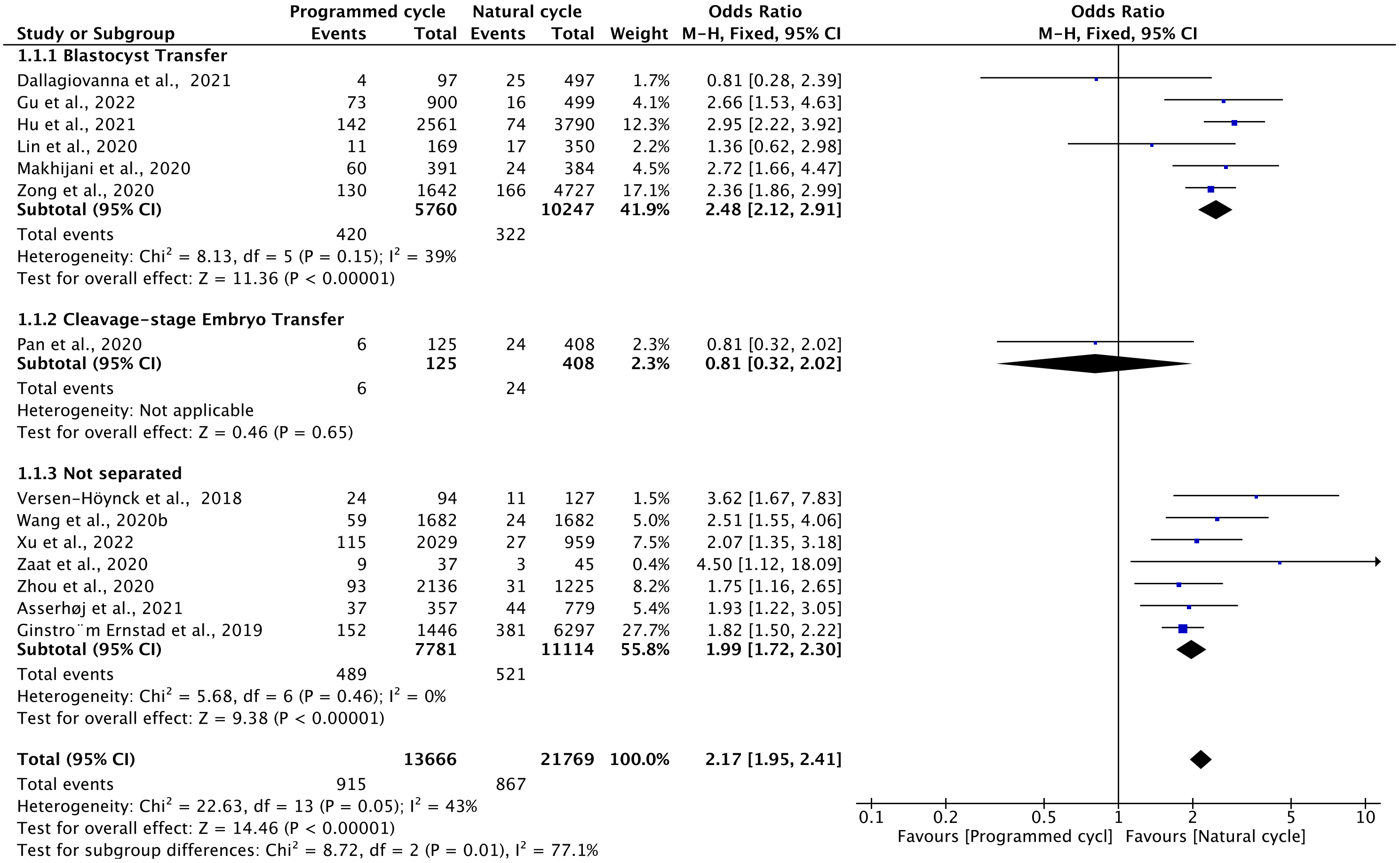

Study or Subgroup	Programmed cycle Events	Programmed cycle Total	Natural cycle Events	Natural cycle Total	Weight	Odds Ratio M-H, Fixed, 95% CI
**1.2.1 Blastocyst Transfer**						
Dallagiovanna et al., 2021	3	97	19	497	3.1%	0.80 [0.23, 2.77]
Fu et al., 2022	33	1304	3	427	2.2%	3.67 [1.12, 12.03]
Gu et al., 2022	42	900	9	499	5.6%	2.67 [1.29, 5.52]
Li et al., 2022	66	1269	7	219	5.7%	1.66 [0.75, 3.67]
Man et al., 2020	55	701	7	102	5.7%	1.16 [0.51, 2.61]
**Subtotal (95% CI)**		**4271**		**1744**	**22.4%**	**1.87 [1.27, 2.75]**
Total events	199		45			
Heterogeneity: $Chi^2 = 5.36$, df = 4 (P = 0.25); $I^2 = 25\%$						
Test for overall effect: Z = 3.17 (P = 0.002)						
**1.2.2 Cleavage-stage Embryo Transfer**						
Guan et al., 2016	20	351	13	215	7.7%	0.94 [0.46, 1.93]
Li et al., 2022	21	457	6	95	4.8%	0.71 [0.28, 1.82]
**Subtotal (95% CI)**		**808**		**310**	**12.5%**	**0.85 [0.48, 1.51]**
Total events	41		19			
Heterogeneity: $Chi^2 = 0.21$, df = 1 (P = 0.65); $I^2 = 0\%$						
Test for overall effect: Z = 0.54 (P = 0.59)						
**1.2.3 Not separated**						
Li et al., 2021	90	1234	91	1491	38.8%	1.21 [0.90, 1.64]
Li et al., 2022	48	1171	7	210	5.8%	1.24 [0.55, 2.78]
Zaat et al., 2020	6	37	2	45	0.8%	4.16 [0.79, 22.01]
Ginstro¨m Ernstad et al., 2019	35	1446	107	6297	19.8%	1.43 [0.98, 2.11]
**Subtotal (95% CI)**		**3888**		**8043**	**65.1%**	**1.32 [1.05, 1.65]**
Total events	179		207			
Heterogeneity: $Chi^2 = 2.35$, df = 3 (P = 0.50); $I^2 = 0\%$						
Test for overall effect: Z = 2.38 (P = 0.02)						
**Total (95% CI)**		**8967**		**10097**	**100.0%**	**1.38 [1.15, 1.66]**
Total events	419		271			
Heterogeneity: $Chi^2 = 12.40$, df = 10 (P = 0.26); $I^2 = 19\%$						
Test for overall effect: Z = 3.45 (P = 0.0006)						
Test for subgroup differences: $Chi^2 = 5.22$, df = 2 (P = 0.07), $I^2 = 61.7\%$						

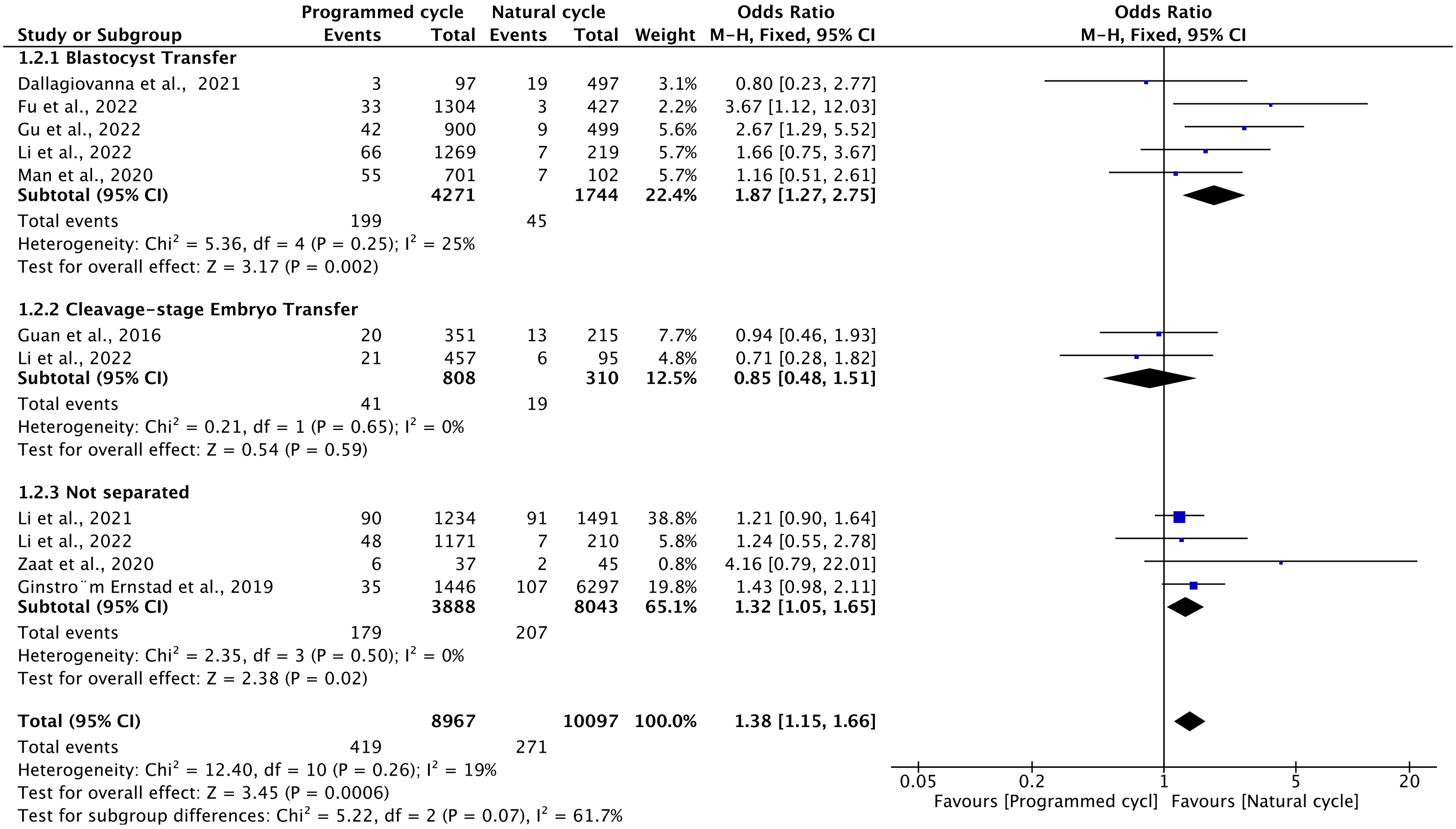

Study or Subgroup	Programmed cycle Events	Programmed cycle Total	Natural cycle Events	Natural cycle Total	Weight	Odds Ratio M-H, Fixed, 95% CI
**1.3.1 Blastocyst Transfer**						
Dallagiovanna et al., 2021	1	97	6	497	0.4%	0.85 [0.10, 7.16]
Gu et al., 2022	31	900	7	499	1.9%	2.51 [1.10, 5.74]
Li et al., 2022	118	1269	14	219	4.6%	1.50 [0.85, 2.66]
Lin et al., 2020	6	169	9	350	1.2%	1.39 [0.49, 3.98]
Man et al., 2020	5	701	1	102	0.4%	0.73 [0.08, 6.27]
Wang et al., 2020a	356	4162	393	10211	44.3%	2.34 [2.01, 2.71]
**Subtotal (95% CI)**		**7298**		**11878**	**52.8%**	**2.23 [1.93, 2.56]**
Total events	517		430			
Heterogeneity: $Chi^2 = 4.89$, df = 5 (P = 0.43); $I^2 = 0\%$						
Test for overall effect: Z = 11.14 (P < 0.00001)						
**1.3.2 Cleavage-stage Embryo Transfer**						
Li et al., 2022	56	457	10	95	3.1%	1.19 [0.58, 2.42]
**Subtotal (95% CI)**		**457**		**95**	**3.1%**	**1.19 [0.58, 2.42]**
Total events	56		10			
Heterogeneity: Not applicable						
Test for overall effect: Z = 0.47 (P = 0.64)						
**1.3.3 Not separated**						
Li et al., 2021	146	1234	99	1491	16.8%	1.89 [1.44, 2.46]
Li et al., 2022	78	1171	8	210	2.7%	1.80 [0.86, 3.79]
Versen-Höynck et al., 2018	12	94	5	127	0.8%	3.57 [1.21, 10.52]
Asserhøj et al., 2021	33	357	31	779	3.8%	2.46 [1.48, 4.08]
Ginstro¨m Ernstad et al., 2019	118	1446	274	6297	20.0%	1.95 [1.56, 2.44]
**Subtotal (95% CI)**		**4302**		**8904**	**44.1%**	**1.99 [1.70, 2.33]**
Total events	387		417			
Heterogeneity: $Chi^2 = 2.04$, df = 4 (P = 0.73); $I^2 = 0\%$						
Test for overall effect: Z = 8.55 (P < 0.00001)						
**Total (95% CI)**		**12057**		**20877**	**100.0%**	**2.09 [1.88, 2.32]**
Total events	960		857			
Heterogeneity: $Chi^2 = 10.65$, df = 11 (P = 0.47); $I^2 = 0\%$						
Test for overall effect: Z = 13.88 (P < 0.00001)						
Test for subgroup differences: $Chi^2 = 3.55$, df = 2 (P = 0.17), $I^2 = 43.7\%$						

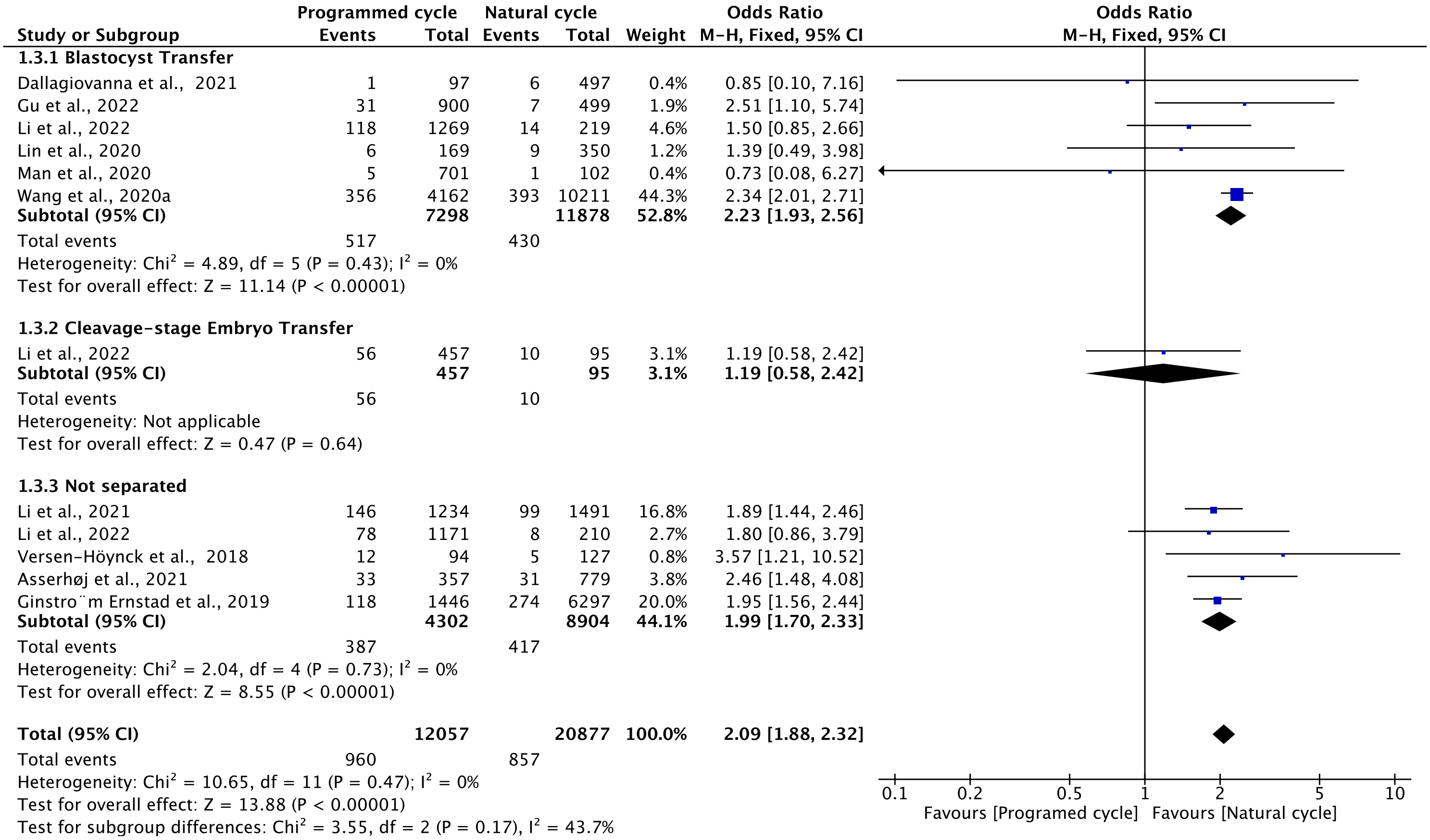

Study or Subgroup	Programmed cycle Events	Programmed cycle Total	Natural cycle Events	Natural cycle Total	Weight	Odds Ratio M-H, Fixed, 95% CI
**1.4.1 Blastocyst Transfer**						
Dallagiovanna et al., 2021	13	97	28	497	0.5%	2.59 [1.29, 5.21]
Fu et al., 2022	121	1304	27	427	2.4%	1.52 [0.98, 2.33]
Gu et al., 2022	206	900	120	499	7.9%	0.94 [0.72, 1.21]
Hu et al., 2021	261	2561	346	3790	16.6%	1.13 [0.95, 1.34]
Li et al., 2022	384	1269	72	219	5.7%	0.89 [0.65, 1.20]
Lin et al., 2020	19	169	40	350	1.5%	0.98 [0.55, 1.75]
Makhijani et al., 2020	42	391	28	384	1.7%	1.53 [0.93, 2.52]
Man et al., 2020	11	701	0	102	0.1%	3.41 [0.20, 58.38]
Wang et al., 2020a	259	4162	575	10211	20.7%	1.11 [0.96, 1.29]
Zong et al., 2020	106	1642	247	4727	7.9%	1.25 [0.99, 1.58]
**Subtotal (95% CI)**		**13196**		**21206**	**65.1%**	**1.13 [1.04, 1.23]**
Total events	1422		1483			
Heterogeneity: $Chi^2 = 14.65$, df = 9 (P = 0.10); $I^2 = 39\%$						
Test for overall effect: Z = 2.79 (P = 0.005)						
**1.4.2 Cleavage-stage Embryo Transfer**						
Guan et al., 2016	5	351	5	215	0.4%	0.61 [0.17, 2.12]
Li et al., 2022	153	457	33	95	2.4%	0.95 [0.59, 1.51]
Pan et al., 2020	1	125	16	408	0.5%	0.20 [0.03, 1.50]
**Subtotal (95% CI)**		**933**		**718**	**3.3%**	**0.79 [0.52, 1.20]**
Total events	159		54			
Heterogeneity: $Chi^2 = 2.53$, df = 2 (P = 0.28); $I^2 = 21\%$						
Test for overall effect: Z = 1.10 (P = 0.27)						
**1.4.3 Not separated**						
Li et al., 2021	124	1234	140	1491	7.6%	1.08 [0.84, 1.39]
Li et al., 2022	390	1171	69	210	5.2%	1.02 [0.75, 1.39]
Versen-Höynck et al., 2018	23	94	27	127	1.2%	1.20 [0.64, 2.26]
Wang et al., 2020b	138	1682	131	1682	8.0%	1.06 [0.82, 1.36]
Xu et al., 2022	28	2029	11	959	1.0%	1.21 [0.60, 2.43]
Zaat et al., 2020	1	37	1	45	0.1%	1.22 [0.07, 20.23]
Zhou et al., 2020	191	2136	113	1225	8.7%	0.97 [0.76, 1.23]
**Subtotal (95% CI)**		**8383**		**5739**	**31.6%**	**1.04 [0.92, 1.18]**
Total events	895		492			
Heterogeneity: $Chi^2 = 0.84$, df = 6 (P = 0.99); $I^2 = 0\%$						
Test for overall effect: Z = 0.63 (P = 0.53)						
**Total (95% CI)**		**22512**		**27663**	**100.0%**	**1.09 [1.02, 1.17]**
Total events	2476		2029			
Heterogeneity: $Chi^2 = 20.57$, df = 19 (P = 0.36); $I^2 = 8\%$						
Test for overall effect: Z = 2.43 (P = 0.02)						
Test for subgroup differences: $Chi^2 = 3.45$, df = 2 (P = 0.18), $I^2 = 42.0\%$						

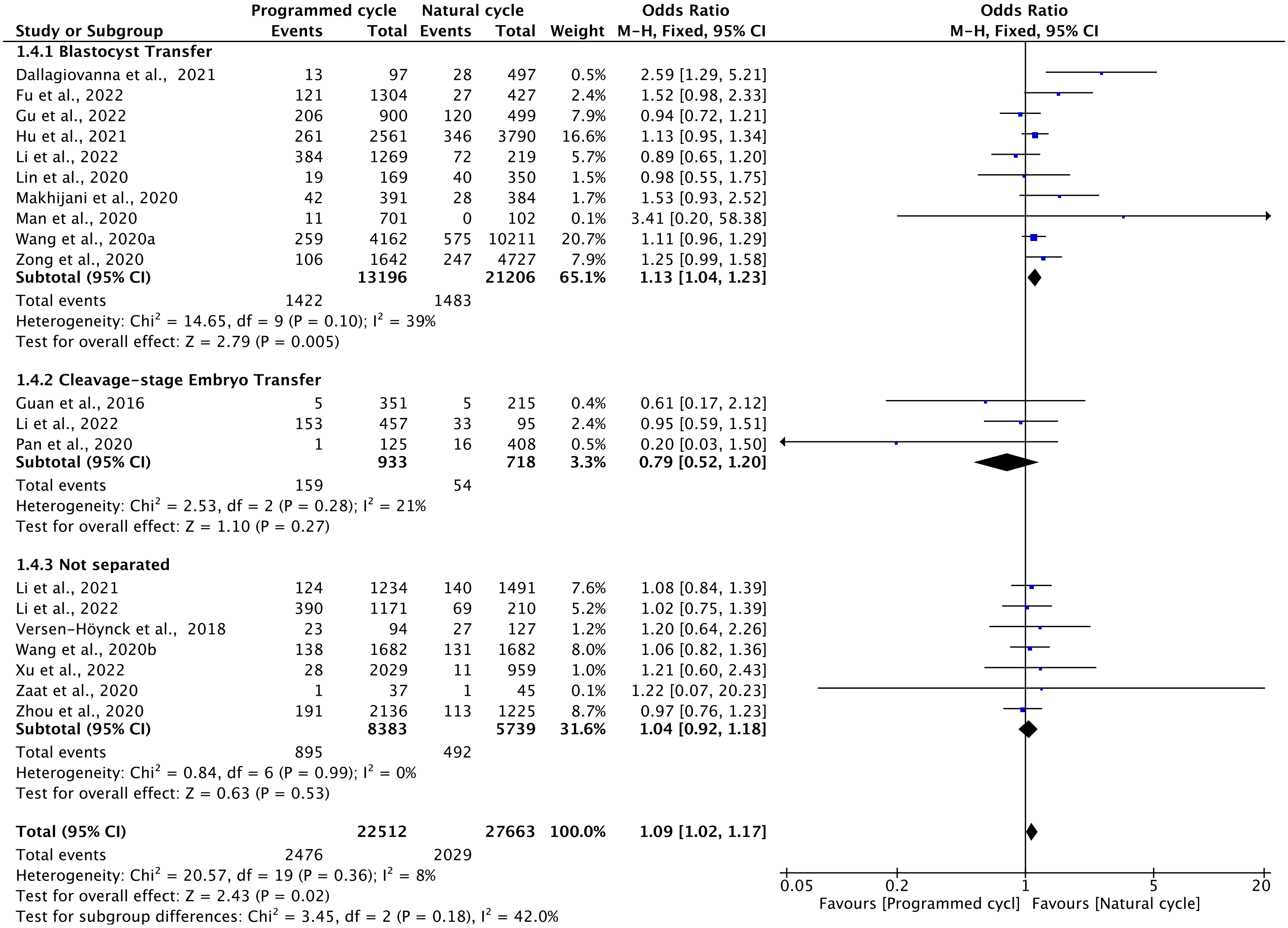

Study or Subgroup	Programmed cycle Events	Programmed cycle Total	Natural cycle Events	Natural cycle Total	Weight	Odds Ratio M-H, Fixed, 95% CI
**1.13.1 Blastocyst Transfer**						
Fu et al., 2022	104	1116	29	364	0.8%	1.19 [0.77, 1.82]
Gu et al., 2022	81	900	29	499	0.7%	1.60 [1.03, 2.49]
Hu et al., 2021	614	2561	784	3790	9.4%	1.21 [1.07, 1.36]
Lin et al., 2020	27	144	58	310	0.6%	1.00 [0.60, 1.66]
Wang et al., 2020a	855	4162	1987	10211	17.8%	1.07 [0.98, 1.17]
Zong et al., 2020	430	1642	1104	4727	8.2%	1.16 [1.02, 1.32]
**Subtotal (95% CI)**		**10525**		**19901**	**37.4%**	**1.14 [1.07, 1.21]**
Total events	2111		3991			
Heterogeneity: $Chi^2 = 5.52$, df = 5 (P = 0.36); $I^2 = 9\%$						
Test for overall effect: Z = 4.11 (P < 0.0001)						
**1.13.2 Cleavage-stage Embryo Transfer**						
Pan et al., 2020	15	144	48	521	0.4%	1.15 [0.62, 2.11]
**Subtotal (95% CI)**		**144**		**521**	**0.4%**	**1.15 [0.62, 2.11]**
Total events	15		48			
Heterogeneity: Not applicable						
Test for overall effect: Z = 0.44 (P = 0.66)						
**1.13.3 Not separated**						
Jing et al., 2019	222	787	747	2802	4.6%	1.08 [0.91, 1.29]
Li et al., 2021	251	1234	350	1491	4.9%	0.83 [0.69, 1.00]
Li et al., 2022	358	1171	50	210	1.1%	1.41 [1.00, 1.98]
Saito et al., 2017	1456	10002	870	6238	17.8%	1.05 [0.96, 1.15]
Tatsumi et al., 2017	1431	14549	996	10526	20.3%	1.04 [0.96, 1.14]
Wang et al., 2020b	335	1682	285	1682	4.4%	1.22 [1.02, 1.45]
Xu et al., 2022	387	2029	144	959	3.1%	1.33 [1.08, 1.64]
Zaat et al., 2020	11	37	13	45	0.2%	1.04 [0.40, 2.71]
Zhou et al., 2020	298	2136	126	1225	2.7%	1.41 [1.13, 1.76]
Asserhøj et al., 2021	21	357	40	779	0.5%	1.15 [0.67, 1.99]
Ginstro¨m Ernstad et al., 2019	112	1446	386	6297	2.6%	1.29 [1.03, 1.60]
**Subtotal (95% CI)**		**35430**		**32254**	**62.2%**	**1.09 [1.04, 1.15]**
Total events	4882		4007			
Heterogeneity: $Chi^2 = 24.84$, df = 10 (P = 0.006); $I^2 = 60\%$						
Test for overall effect: Z = 3.61 (P = 0.0003)						
**Total (95% CI)**		**46099**		**52676**	**100.0%**	**1.11 [1.07, 1.15]**
Total events	7008		8046			
Heterogeneity: $Chi^2 = 31.36$, df = 17 (P = 0.02); $I^2 = 46\%$						
Test for overall effect: Z = 5.38 (P < 0.00001)						
Test for subgroup differences: $Chi^2 = 1.00$, df = 2 (P = 0.61), $I^2 = 0\%$						

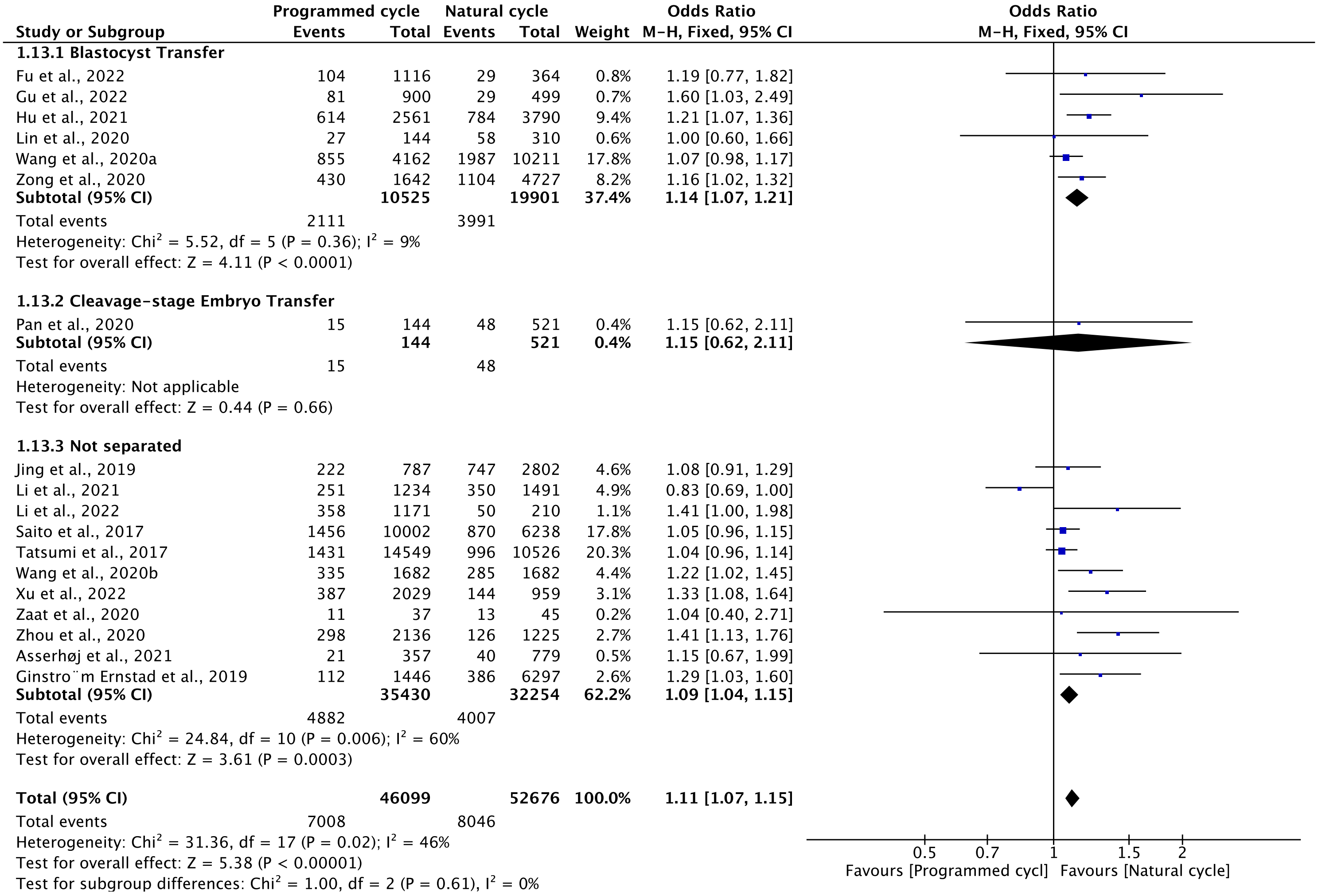

Study or Subgroup	Programmed cycle Events	Programmed cycle Total	Natural cycle Events	Natural cycle Total	Weight	Odds Ratio M-H, Fixed, 95% CI
**1.6.1 Blastocyst Transfer**						
Gu et al., 2022	39	900	12	499	1.2%	1.84 [0.95, 3.54]
Hu et al., 2021	333	2561	390	3790	21.4%	1.30 [1.12, 1.52]
Makhijani et al., 2020	54	386	36	381	2.4%	1.56 [1.00, 2.44]
Wang et al., 2020a	433	4162	1032	10211	41.9%	1.03 [0.92, 1.16]
**Subtotal (95% CI)**		**8009**		**14881**	**66.9%**	**1.15 [1.05, 1.26]**
Total events	859		1470			
Heterogeneity: $Chi^2 = 9.37$, df = 3 (P = 0.02); $I^2 = 68\%$						
Test for overall effect: Z = 3.06 (P = 0.002)						
**1.6.2 Cleavage-stage Embryo Transfer**						
Pan et al., 2020	8	144	24	521	0.8%	1.22 [0.54, 2.77]
**Subtotal (95% CI)**		**144**		**521**	**0.8%**	**1.22 [0.54, 2.77]**
Total events	8		24			
Heterogeneity: Not applicable						
Test for overall effect: Z = 0.47 (P = 0.64)						
**1.6.3 Not separated**						
Jing et al., 2019	50	787	196	2802	6.3%	0.90 [0.65, 1.24]
Li et al., 2021	91	1234	104	1491	6.8%	1.06 [0.79, 1.42]
Li et al., 2022	137	1171	24	210	2.8%	1.03 [0.65, 1.63]
Saito et al., 2017	144	10209	67	6272	6.4%	1.32 [0.99, 1.77]
Wang et al., 2020b	136	1682	112	1682	8.1%	1.23 [0.95, 1.60]
Xu et al., 2022	45	2029	15	959	1.6%	1.43 [0.79, 2.57]
Zaat et al., 2020	11	37	8	54	0.4%	2.43 [0.87, 6.81]
**Subtotal (95% CI)**		**17149**		**13470**	**32.3%**	**1.16 [1.01, 1.32]**
Total events	614		526			
Heterogeneity: $Chi^2 = 6.45$, df = 6 (P = 0.38); $I^2 = 7\%$						
Test for overall effect: Z = 2.15 (P = 0.03)						
**Total (95% CI)**		**25302**		**28872**	**100.0%**	**1.15 [1.07, 1.24]**
Total events	1481		2020			
Heterogeneity: $Chi^2 = 15.83$, df = 11 (P = 0.15); $I^2 = 31\%$						
Test for overall effect: Z = 3.77 (P = 0.0002)						
Test for subgroup differences: $Chi^2 = 0.02$, df = 2 (P = 0.99), $I^2 = 0\%$						

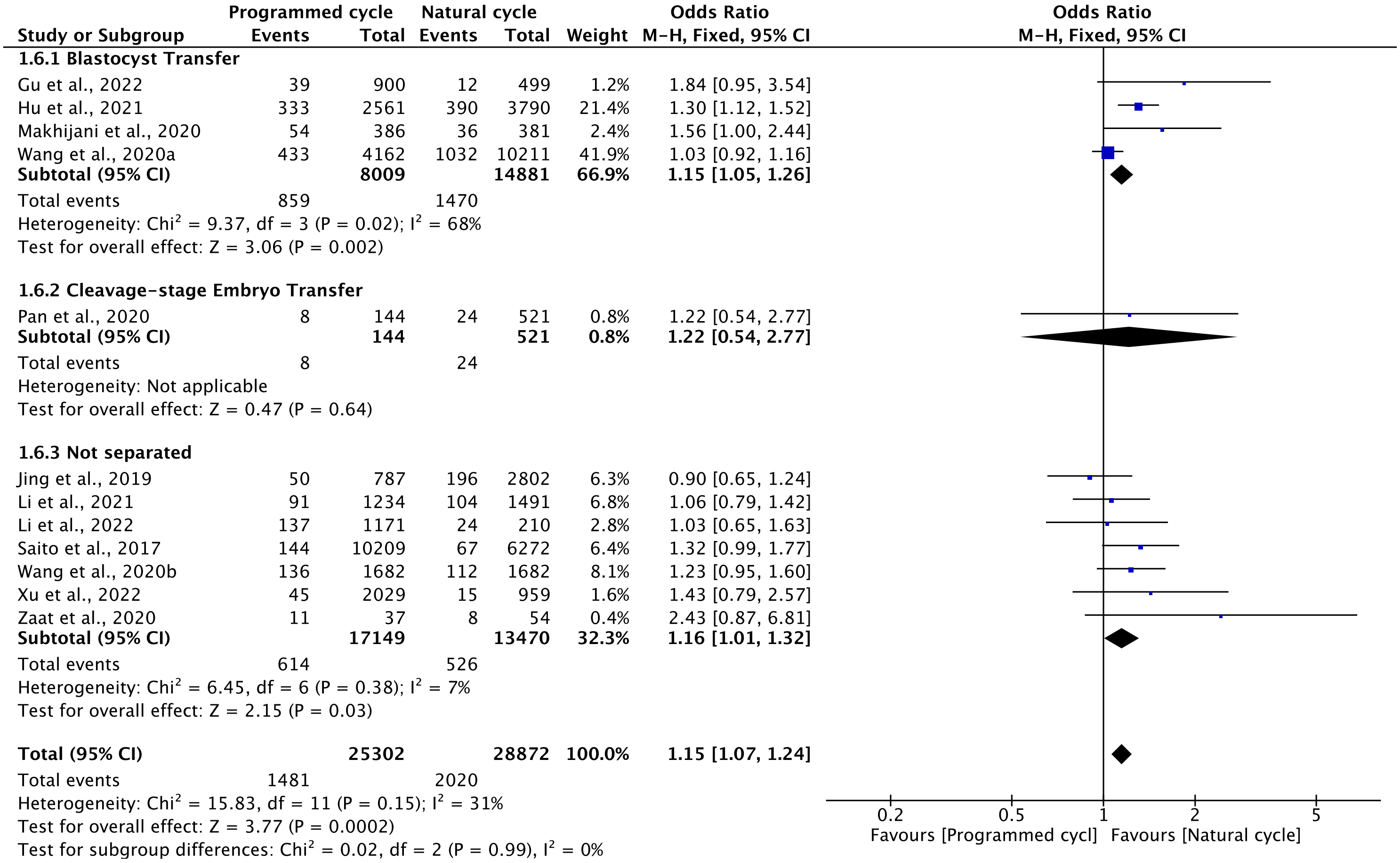

Study or Subgroup	Programmed cycle Events	Programmed cycle Total	Natural cycle Events	Natural cycle Total	Weight	Odds Ratio M-H, Fixed, 95% CI
**1.7.1 Blastocyst Transfer**						
Dallagiovanna et al., 2021	6	97	31	497	0.3%	0.99 [0.40, 2.44]
Fu et al., 2022	103	1116	25	364	1.2%	1.38 [0.88, 2.17]
Gu et al., 2022	116	900	56	499	2.2%	1.17 [0.83, 1.64]
Hu et al., 2021	303	2561	292	3790	7.4%	1.61 [1.36, 1.90]
Lin et al., 2020	10	169	22	350	0.5%	0.94 [0.43, 2.03]
Makhijani et al., 2020	51	391	48	384	1.5%	1.05 [0.69, 1.60]
Man et al., 2020	67	701	5	102	0.3%	2.05 [0.81, 5.21]
Wang et al., 2020a	315	4162	581	10211	11.0%	1.36 [1.18, 1.56]
Zong et al., 2020	129	1642	227	4727	3.8%	1.69 [1.35, 2.11]
**Subtotal (95% CI)**		**11739**		**20924**	**28.3%**	**1.43 [1.31, 1.57]**
Total events	1100		1287			
Heterogeneity: $Chi^2 = 10.27$, df = 8 (P = 0.25); $I^2 = 22\%$						
Test for overall effect: Z = 7.96 (P < 0.00001)						
**1.7.2 Cleavage-stage Embryo Transfer**						
Guan et al., 2016	60	271	39	184	1.3%	1.06 [0.67, 1.67]
Pan et al., 2020	20	110	63	357	0.9%	1.04 [0.59, 1.81]
**Subtotal (95% CI)**		**381**		**541**	**2.1%**	**1.05 [0.74, 1.49]**
Total events	80		102			
Heterogeneity: $Chi^2 = 0.00$, df = 1 (P = 0.96); $I^2 = 0\%$						
Test for overall effect: Z = 0.27 (P = 0.79)						
**1.7.3 Not separated**						
Jing et al., 2019	61	787	202	2802	2.9%	1.08 [0.80, 1.46]
Li et al., 2021	170	1234	181	1491	5.0%	1.16 [0.92, 1.45]
Li et al., 2022	111	1171	22	210	1.2%	0.89 [0.55, 1.45]
Saito et al., 2017	737	10235	411	6287	16.8%	1.11 [0.98, 1.26]
Tatsumi et al., 2017	1094	15579	725	10972	28.1%	1.07 [0.97, 1.18]
Versen-Höynck et al., 2018	13	94	14	127	0.4%	1.30 [0.58, 2.90]
Wang et al., 2020b	124	1682	90	1682	3.0%	1.41 [1.06, 1.86]
Xu et al., 2022	159	2029	50	959	2.2%	1.55 [1.11, 2.14]
Zhou et al., 2020	219	2136	102	1225	4.1%	1.26 [0.98, 1.61]
Asserhøj et al., 2021	26	357	64	779	1.3%	0.88 [0.55, 1.41]
Ginstro¨m Ernstad et al., 2019	98	1446	377	6297	4.7%	1.14 [0.91, 1.44]
**Subtotal (95% CI)**		**36750**		**32831**	**69.6%**	**1.13 [1.06, 1.20]**
Total events	2812		2238			
Heterogeneity: $Chi^2 = 10.21$, df = 10 (P = 0.42); $I^2 = 2\%$						
Test for overall effect: Z = 3.79 (P = 0.0001)						
**Total (95% CI)**		**48870**		**54296**	**100.0%**	**1.21 [1.15, 1.27]**
Total events	3992		3627			
Heterogeneity: $Chi^2 = 40.99$, df = 21 (P = 0.006); $I^2 = 49\%$						
Test for overall effect: Z = 7.52 (P < 0.00001)						
Test for subgroup differences: $Chi^2 = 20.10$, df = 2 (P < 0.0001), $I^2 = 90.1\%$						

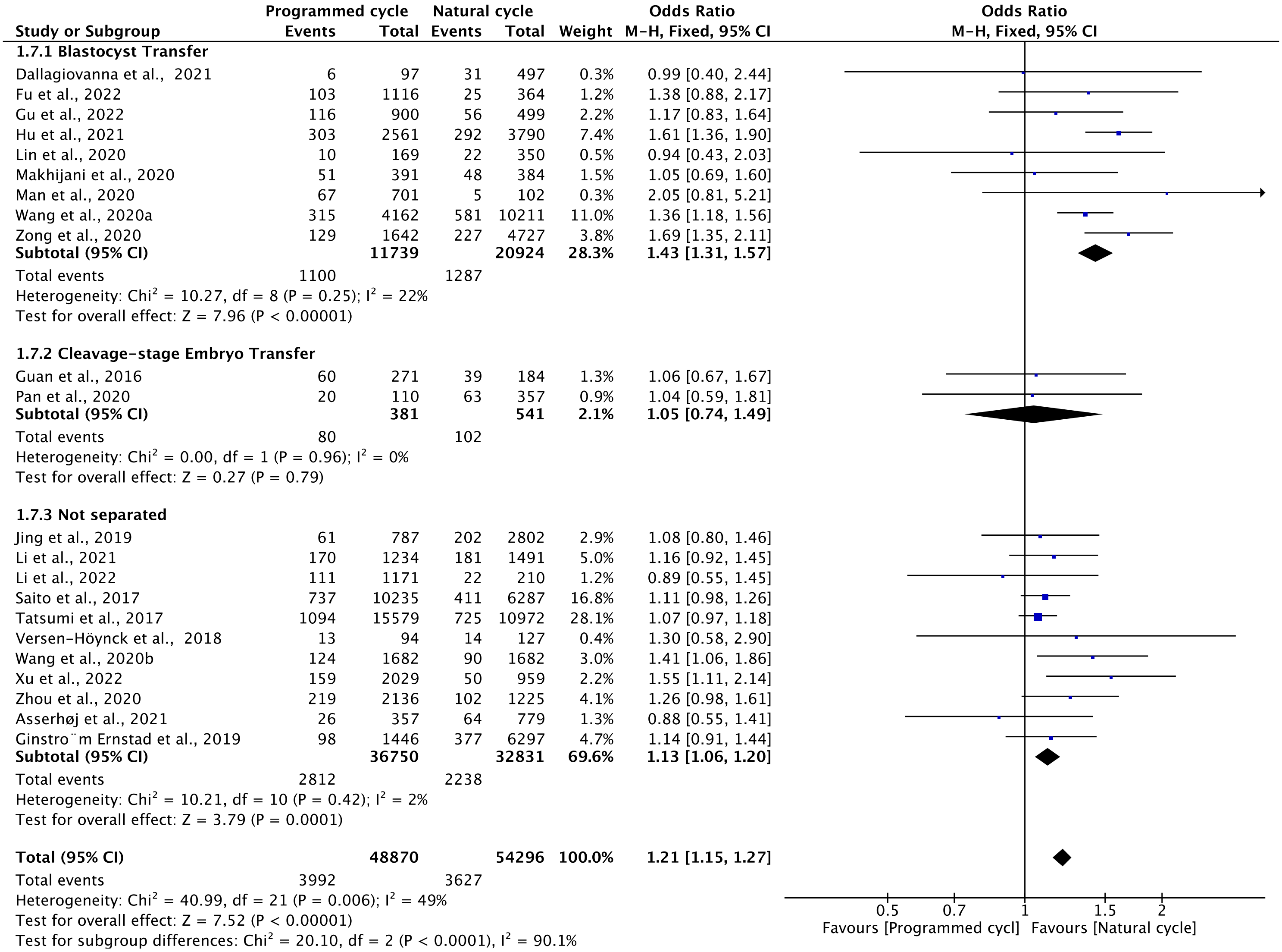

Study or Subgroup	Programmed cycle Events	Programmed cycle Total	Natural cycle Events	Natural cycle Total	Weight	Odds Ratio M-H, Fixed, 95% CI
**1.10.1 Blastocyst Transfer**						
Fu et al., 2022	81	1116	27	364	1.7%	0.98 [0.62, 1.54]
Gu et al., 2022	68	900	39	499	2.1%	0.96 [0.64, 1.45]
Hu et al., 2021	88	2561	141	3790	5.0%	0.92 [0.70, 1.21]
Lin et al., 2020	10	144	16	310	0.4%	1.37 [0.61, 3.10]
Wang et al., 2020a	226	4162	561	10211	14.1%	0.99 [0.84, 1.16]
Zong et al., 2020	48	1642	165	4727	3.8%	0.83 [0.60, 1.15]
**Subtotal (95% CI)**		**10525**		**19901**	**27.2%**	**0.96 [0.85, 1.07]**
Total events	521		949			
Heterogeneity: $Chi^2 = 1.68$, df = 5 (P = 0.89); $I^2 = 0\%$						
Test for overall effect: Z = 0.74 (P = 0.46)						
**1.10.2 Cleavage-stage Embryo Transfer**						
Pan et al., 2020	18	144	75	521	1.3%	0.85 [0.49, 1.47]
**Subtotal (95% CI)**		**144**		**521**	**1.3%**	**0.85 [0.49, 1.47]**
Total events	18		75			
Heterogeneity: Not applicable						
Test for overall effect: Z = 0.58 (P = 0.56)						
**1.10.3 Not separated**						
Jing et al., 2019	30	787	85	2802	1.6%	1.27 [0.83, 1.94]
Li et al., 2021	126	1234	104	1491	3.9%	1.52 [1.16, 1.99]
Li et al., 2022	55	1171	11	210	0.8%	0.89 [0.46, 1.73]
Saito et al., 2017	609	10002	323	6238	17.1%	1.19 [1.03, 1.36]
Tatsumi et al., 2017	986	14549	667	10526	33.1%	1.07 [0.97, 1.19]
Wang et al., 2020b	71	1682	76	1682	3.3%	0.93 [0.67, 1.30]
Xu et al., 2022	86	2029	54	959	3.2%	0.74 [0.52, 1.05]
Zaat et al., 2020	1	37	3	45	0.1%	0.39 [0.04, 3.90]
Zhou et al., 2020	119	2136	86	1225	4.7%	0.78 [0.59, 1.04]
Asserhøj et al., 2021	14	357	20	779	0.6%	1.55 [0.77, 3.10]
Ginstro¨m Ernstad et al., 2019	39	1446	179	6297	3.0%	0.95 [0.67, 1.35]
**Subtotal (95% CI)**		**35430**		**32254**	**71.5%**	**1.08 [1.01, 1.16]**
Total events	2136		1608			
Heterogeneity: $Chi^2 = 21.10$, df = 10 (P = 0.02); $I^2 = 53\%$						
Test for overall effect: Z = 2.29 (P = 0.02)						
**Total (95% CI)**		**46099**		**52676**	**100.0%**	**1.05 [0.99, 1.11]**
Total events	2675		2632			
Heterogeneity: $Chi^2 = 26.52$, df = 17 (P = 0.07); $I^2 = 36\%$						
Test for overall effect: Z = 1.52 (P = 0.13)						
Test for subgroup differences: $Chi^2 = 3.84$, df = 2 (P = 0.15), $I^2 = 48.0\%$						

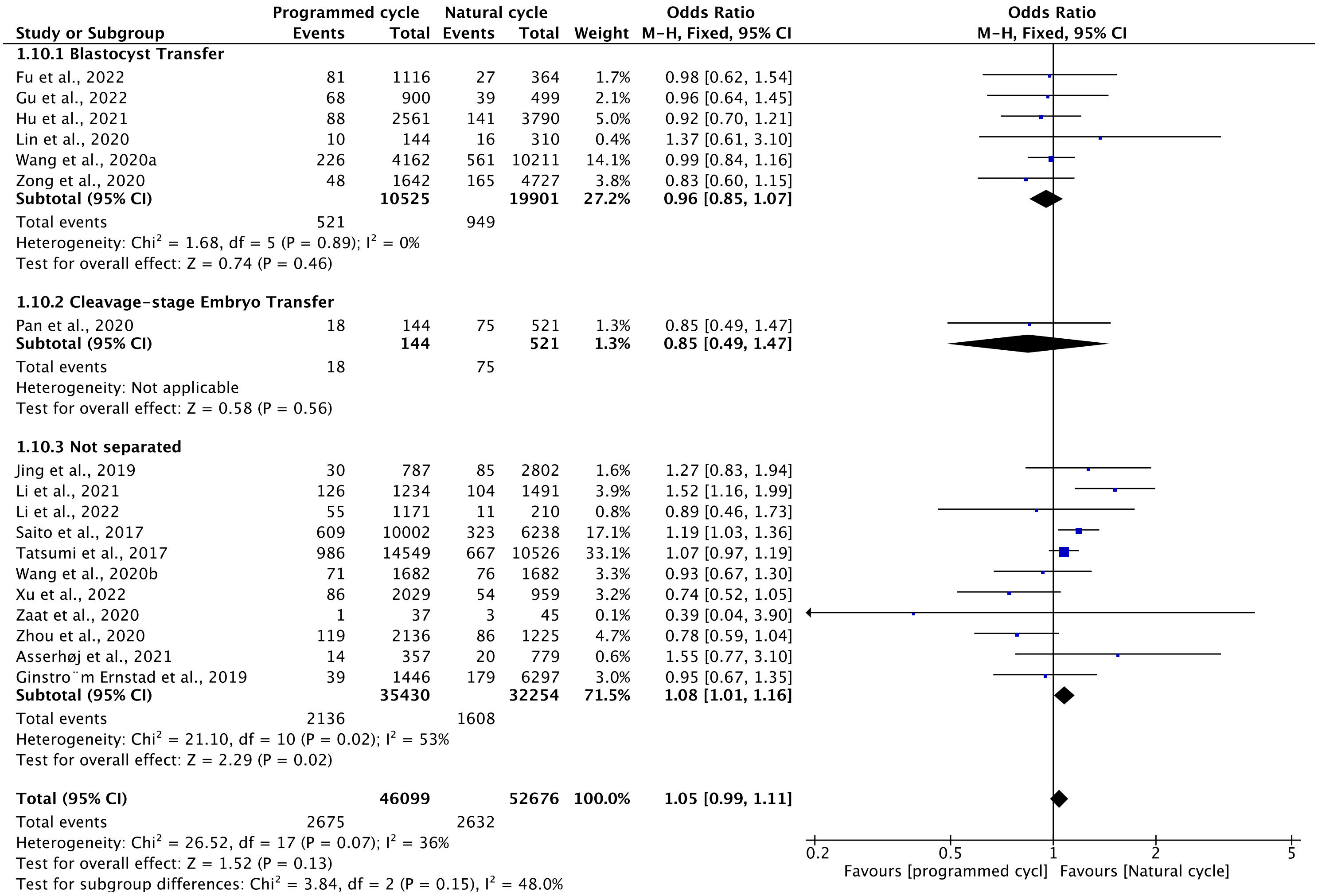

Study or Subgroup	Programmed cycle Events	Programmed cycle Total	Natural cycle Events	Natural cycle Total	Weight	Odds Ratio M-H, Fixed, 95% CI
**1.11.1 Blastocyst Transfer**						
Dallagiovanna et al., 2021	1	97	8	497	1.0%	0.64 [0.08, 5.15]
Gu et al., 2022	44	900	16	499	7.4%	1.55 [0.87, 2.78]
Hu et al., 2021	14	2561	15	3790	4.5%	1.38 [0.67, 2.87]
Li et al., 2022	42	1269	12	219	7.5%	0.59 [0.31, 1.14]
Lin et al., 2020	0	18	6	350	0.2%	1.43 [0.08, 26.41]
Makhijani et al., 2020	10	391	9	384	3.3%	1.09 [0.44, 2.72]
Man et al., 2020	4	701	0	102	0.3%	1.32 [0.07, 24.75]
Wang et al., 2020a	39	4162	77	10211	16.7%	1.24 [0.85, 1.83]
Zong et al., 2020	24	1642	47	4727	9.0%	1.48 [0.90, 2.42]
**Subtotal (95% CI)**		**11741**		**20779**	**50.0%**	**1.23 [0.98, 1.54]**
Total events	178		190			
Heterogeneity: $Chi^2 = 6.47$, df = 8 (P = 0.60); $I^2 = 0\%$						
Test for overall effect: Z = 1.76 (P = 0.08)						
**1.11.2 Cleavage-stage Embryo Transfer**						
Li et al., 2022	42	1269	12	219	7.5%	0.59 [0.31, 1.14]
**Subtotal (95% CI)**		**1269**		**219**	**7.5%**	**0.59 [0.31, 1.14]**
Total events	42		12			
Heterogeneity: Not applicable						
Test for overall effect: Z = 1.57 (P = 0.12)						
**1.11.3 Not separated**						
Li et al., 2022	43	1171	8	210	4.9%	0.96 [0.45, 2.08]
Versen-Höynck et al., 2018	3	94	5	127	1.6%	0.80 [0.19, 3.45]
Wang et al., 2020b	66	1682	39	1682	14.2%	1.72 [1.15, 2.57]
Xu et al., 2022	49	2029	15	959	7.5%	1.56 [0.87, 2.79]
Zaat et al., 2020	1	37	2	45	0.7%	0.60 [0.05, 6.86]
Asserhøj et al., 2021	7	357	15	779	3.5%	1.02 [0.41, 2.52]
Ginstro¨m Ernstad et al., 2019	13	1446	73	6297	10.2%	0.77 [0.43, 1.40]
**Subtotal (95% CI)**		**6816**		**10099**	**42.5%**	**1.27 [0.99, 1.62]**
Total events	182		157			
Heterogeneity: $Chi^2 = 6.82$, df = 6 (P = 0.34); $I^2 = 12\%$						
Test for overall effect: Z = 1.88 (P = 0.06)						
**Total (95% CI)**		**19826**		**31097**	**100.0%**	**1.20 [1.02, 1.41]**
Total events	402		359			
Heterogeneity: $Chi^2 = 17.93$, df = 16 (P = 0.33); $I^2 = 11\%$						
Test for overall effect: Z = 2.16 (P = 0.03)						
Test for subgroup differences: $Chi^2 = 4.67$, df = 2 (P = 0.10), $I^2 = 57.2\%$						

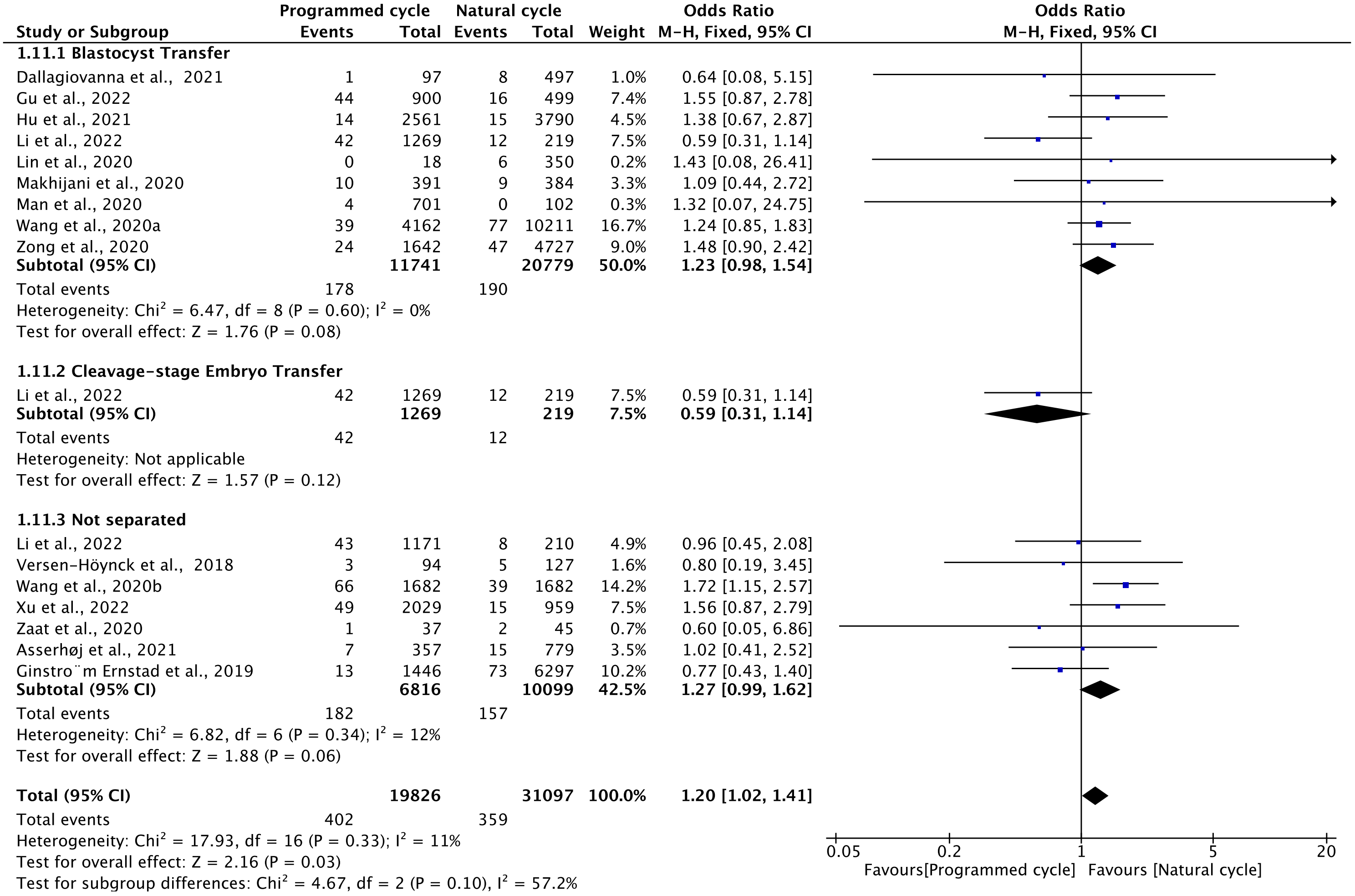

Study or Subgroup	Programmed cycle Events	Programmed cycle Total	Natural cycle Events	Natural cycle Total	Weight	Odds Ratio M-H, Fixed, 95% CI
**1.14.1 Blastocyst Transfer**						
Gu et al., 2022	12	900	8	499	24.9%	0.83 [0.34, 2.04]
Li et al., 2022	5	1269	1	219	4.2%	0.86 [0.10, 7.42]
Makhijani et al., 2020	3	391	2	384	4.9%	1.48 [0.25, 8.89]
Man et al., 2020	2	701	0	102	2.1%	0.73 [0.03, 15.37]
Wang et al., 2020a	9	4162	10	10211	14.2%	2.21 [0.90, 5.44]
**Subtotal (95% CI)**		**7423**		**11415**	**50.3%**	**1.28 [0.72, 2.28]**
Total events	31		21			
Heterogeneity: Chi² = 2.59, df = 4 (P = 0.63); I² = 0%						
Test for overall effect: Z = 0.84 (P = 0.40)						
**1.14.2 Cleavage-stage Embryo Transfer**						
Li et al., 2022	5	1269	1	219	4.2%	0.86 [0.10, 7.42]
**Subtotal (95% CI)**		**1269**		**219**	**4.2%**	**0.86 [0.10, 7.42]**
Total events	5		1			
Heterogeneity: Not applicable						
Test for overall effect: Z = 0.13 (P = 0.89)						
**1.14.3 Not separated**						
Li et al., 2022	3	1117	1	210	4.1%	0.56 [0.06, 5.44]
Versen-Höynck et al., 2018	1	94	0	127	1.0%	4.09 [0.16, 101.54]
Zaat et al., 2020	0	37	1	45	3.3%	0.40 [0.02, 10.00]
Asserhøj et al., 2021	5	357	7	779	10.6%	1.57 [0.49, 4.97]
Ginstro¨m Ernstad et al., 2019	7	1446	29	6297	26.4%	1.05 [0.46, 2.40]
**Subtotal (95% CI)**		**3051**		**7458**	**45.5%**	**1.15 [0.62, 2.12]**
Total events	16		38			
Heterogeneity: Chi² = 1.72, df = 4 (P = 0.79); I² = 0%						
Test for overall effect: Z = 0.44 (P = 0.66)						
**Total (95% CI)**		**11743**		**19092**	**100.0%**	**1.20 [0.80, 1.82]**
Total events	52		60			
Heterogeneity: Chi² = 4.49, df = 10 (P = 0.92); I² = 0%						
Test for overall effect: Z = 0.88 (P = 0.38)						
Test for subgroup differences: Chi² = 0.16, df = 2 (P = 0.92), I² = 0%						

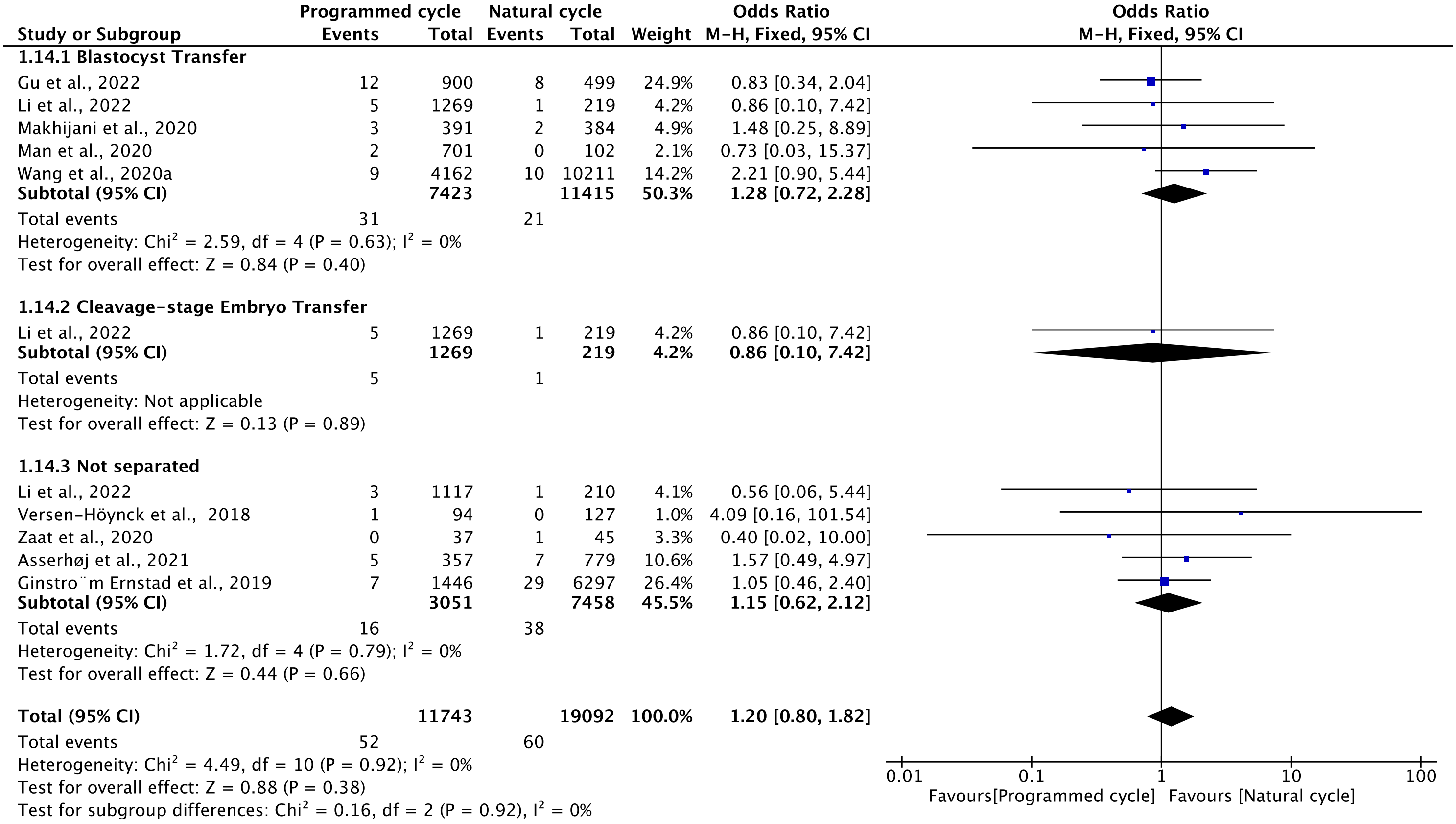

Study or Subgroup	Programmed cycle Events	Total	Natural cycle Events	Total	Weight	Odds Ratio M-H, Fixed, 95% CI
**1.5.1 Blastocyst Transfer**						
Hu et al., 2021	58	2561	52	3790	13.5%	1.67 [1.14, 2.43]
Li et al., 2022	296	1269	42	219	18.0%	1.28 [0.89, 1.84]
Lin et al., 2020	18	169	36	350	6.9%	1.04 [0.57, 1.89]
Makhijani et al., 2020	10	391	1	384	0.3%	10.05 [1.28, 78.91]
**Subtotal (95% CI)**		**4390**		**4743**	**38.7%**	**1.45 [1.14, 1.83]**
Total events	382		131			
Heterogeneity: $Chi^2 = 5.54$, df = 3 (P = 0.14); $I^2 = 46\%$						
Test for overall effect: Z = 3.06 (P = 0.002)						
**1.5.2 Cleavage-stage Embryo Transfer**						
Li et al., 2022	87	457	22	95	9.7%	0.78 [0.46, 1.33]
Pan et al., 2020	3	125	16	408	2.4%	0.60 [0.17, 2.10]
**Subtotal (95% CI)**		**582**		**503**	**12.1%**	**0.74 [0.46, 1.21]**
Total events	90		38			
Heterogeneity: $Chi^2 = 0.14$, df = 1 (P = 0.71); $I^2 = 0\%$						
Test for overall effect: Z = 1.19 (P = 0.23)						
**1.5.3 Not separated**						
Li et al., 2022	271	1171	44	210	18.8%	1.14 [0.79, 1.63]
Xu et al., 2022	63	2029	34	959	14.7%	0.87 [0.57, 1.33]
Asserhøj et al., 2021	50	357	88	779	15.6%	1.28 [0.88, 1.86]
**Subtotal (95% CI)**		**3557**		**1948**	**49.2%**	**1.10 [0.88, 1.38]**
Total events	384		166			
Heterogeneity: $Chi^2 = 1.81$, df = 2 (P = 0.40); $I^2 = 0\%$						
Test for overall effect: Z = 0.86 (P = 0.39)						
**Total (95% CI)**		**8529**		**7194**	**100.0%**	**1.19 [1.02, 1.39]**
Total events	856		335			
Heterogeneity: $Chi^2 = 13.37$, df = 8 (P = 0.10); $I^2 = 40\%$						
Test for overall effect: Z = 2.25 (P = 0.02)						
Test for subgroup differences: $Chi^2 = 6.66$, df = 2 (P = 0.04), $I^2 = 70.0\%$						

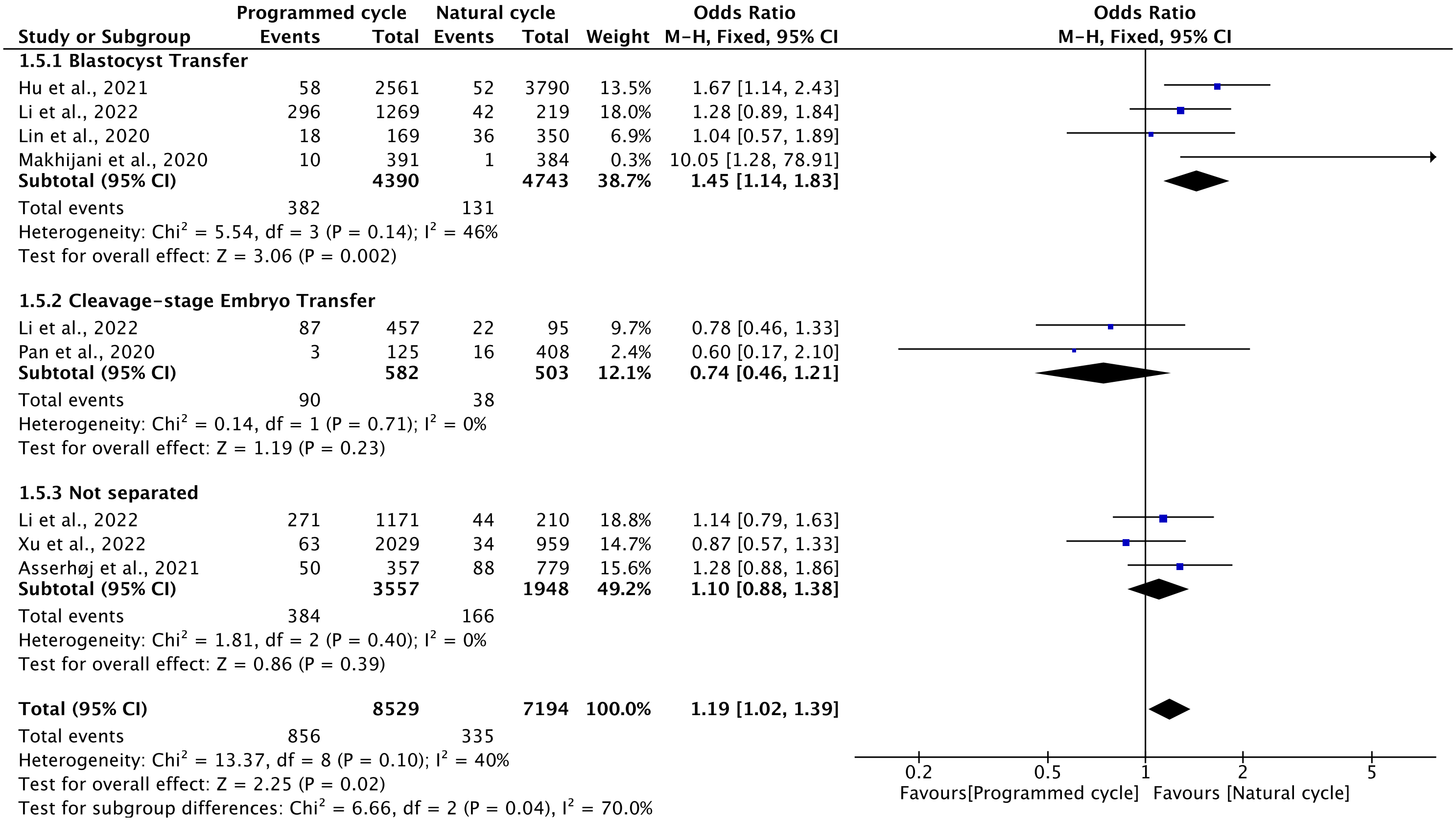

Study or Subgroup	Programmed cycle Events	Programmed cycle Total	Natural cycle Events	Natural cycle Total	Weight	Odds Ratio M-H, Fixed, 95% CI
**1.12.1 Blastocyst Transfer**						
Gu et al., 2022	43	900	18	499	5.9%	1.34 [0.76, 2.35]
Li et al., 2022	308	1269	34	219	11.8%	1.74 [1.18, 2.57]
Lin et al., 2020	5	144	3	308	0.5%	3.66 [0.86, 15.52]
Makhijani et al., 2020	5	391	4	384	1.1%	1.23 [0.33, 4.62]
Man et al., 2020	3	701	1	102	0.5%	0.43 [0.04, 4.21]
Wang et al., 2020a	29	4162	17	10211	2.6%	4.21 [2.31, 7.66]
**Subtotal (95% CI)**		**7567**		**11723**	**22.4%**	**1.92 [1.46, 2.51]**
Total events	393		77			
Heterogeneity: $Chi^2 = 11.23$, df = 5 (P = 0.05); $I^2 = 55\%$						
Test for overall effect: Z = 4.71 (P < 0.00001)						
**1.12.2 Cleavage-stage Embryo Transfer**						
Li et al., 2022	112	457	17	95	5.7%	1.49 [0.85, 2.62]
**Subtotal (95% CI)**		**457**		**95**	**5.7%**	**1.49 [0.85, 2.62]**
Total events	112		17			
Heterogeneity: Not applicable						
Test for overall effect: Z = 1.38 (P = 0.17)						
**1.12.3 Not separated**						
Li et al., 2022	215	1171	26	210	9.7%	1.59 [1.03, 2.46]
Xu et al., 2022	30	2029	6	959	2.2%	2.38 [0.99, 5.75]
Zaat et al., 2020	10	37	6	45	1.1%	2.41 [0.78, 7.41]
Asserhøj et al., 2021	137	357	179	779	18.7%	2.09 [1.59, 2.74]
Ginstro¨m Ernstad et al., 2019	281	1446	497	6297	40.3%	2.81 [2.40, 3.30]
**Subtotal (95% CI)**		**5040**		**8290**	**71.8%**	**2.44 [2.14, 2.78]**
Total events	673		714			
Heterogeneity: $Chi^2 = 8.05$, df = 4 (P = 0.09); $I^2 = 50\%$						
Test for overall effect: Z = 13.34 (P < 0.00001)						
**Total (95% CI)**		**13064**		**20108**	**100.0%**	**2.27 [2.02, 2.55]**
Total events	1178		808			
Heterogeneity: $Chi^2 = 24.57$, df = 11 (P = 0.01); $I^2 = 55\%$						
Test for overall effect: Z = 13.77 (P < 0.00001)						
Test for subgroup differences: $Chi^2 = 4.79$, df = 2 (P = 0.09), $I^2 = 58.3\%$						

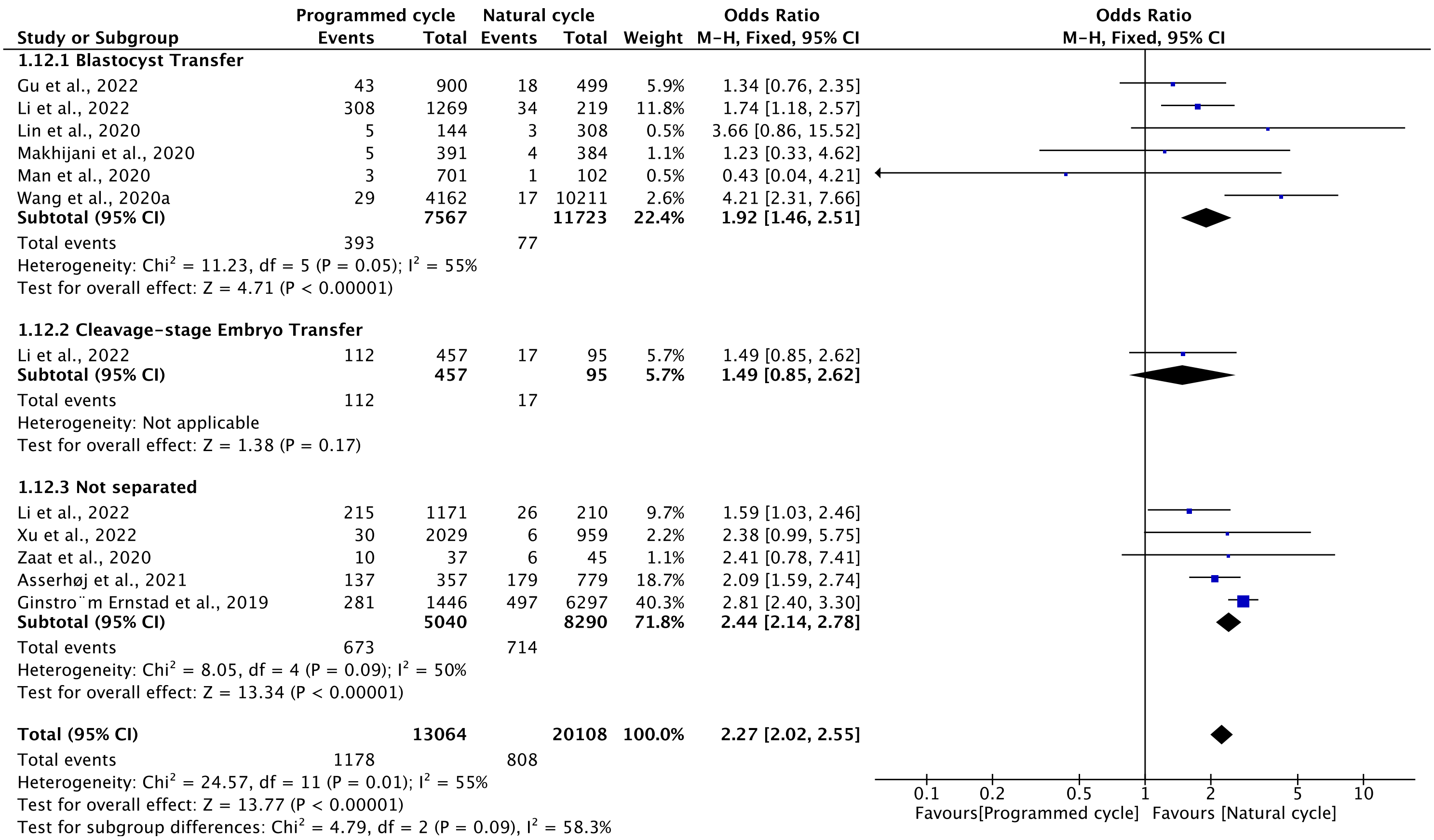

Supplement: Supplementary file 1 [file DataSheet_1.pdf]
